# Deciphering the Molecular Mechanism of Water Interaction with Gelatin Methacryloyl Hydrogels: Role of Ionic Strength, pH, Drug Loading and Hydrogel Network Characteristics

**DOI:** 10.3390/biomedicines9050574

**Published:** 2021-05-19

**Authors:** Margaux Vigata, Christoph Meinert, Nathalie Bock, Bronwin L. Dargaville, Dietmar W. Hutmacher

**Affiliations:** 1 Science and Engineering Faculty (SEF), School of Mechanical, Medical and Process Engineering, Queensland University of Technology (QUT), Brisbane, QLD 4059, Australia; margaux.vigata@hdr.qut.edu.au (M.V.); christoph.meinert@qut.edu.au (C.M.); bronwin.dargaville@qut.edu.au (B.L.D.); 2 Herston Biofabrication Institute, Metro North Hospital and Health Services, Brisbane, QLD 4029, Australia; 3 Faculty of Health, School of Biomedical Sciences, Queensland University of Technology (QUT), Brisbane, QLD 4059, Australia; n.bock@qut.edu.au; 4 Translational Research Institute, Brisbane, QLD 4102, Australia; 5 Australian Research Council Industrial Transformation Training Centre in Additive Biomanufacturing, QUT, Brisbane, QLD 4059, Australia; 6 ARC Industrial Transformation Training Centre (ARC ITTC) for Multiscale 3D Imaging, Modelling and Manufacturing, Brisbane, QLD 4059, Australia

**Keywords:** hydrogel, gelatin methacryloyl, water, swelling, ionic strength, pH, drug, mesh size

## Abstract

Water plays a primary role in the functionality of biomedical polymers such as hydrogels. The state of water, defined as bound, intermediate, or free, and its molecular organization within hydrogels is an important factor governing biocompatibility and hemocompatibility. Here, we present a systematic study of water states in gelatin methacryloyl (GelMA) hydrogels designed for drug delivery and tissue engineering applications. We demonstrate that increasing ionic strength of the swelling media correlated with the proportion of non-freezable bound water. We attribute this to the capability of ions to create ion–dipole bonds with both the polymer and water, thereby reinforcing the first layer of polymer hydration. Both pH and ionic strength impacted the mesh size, having potential implications for drug delivery applications. The mechanical properties of GelMA hydrogels were largely unaffected by variations in ionic strength or pH. Loading of cefazolin, a small polar antibiotic molecule, led to a dose-dependent increase of non-freezable bound water, attributed to the drug’s capacity to form hydrogen bonds with water, which helped recruit water molecules in the hydrogels’ first hydration layer. This work enables a deeper understanding of water states and molecular arrangement at the hydrogel–polymer interface and how environmental cues influence them.

## 1. Introduction

Hydrogels are highly hydrated, hydrophilic, three-dimensional biomaterials commonly applied for tissue engineering, wound dressings, drug delivery, and other applications [1]. The physicochemical and functional properties of hydrogels are largely dependent on their interaction with water [2,3]. This biomaterial class is successfully applied and commercialized for tissue engineering, wound dressing, and drug delivery [1]. Many of the most widely used materials are based on polysaccharide hydrogels such as agarose, chitosan, alginate, and hyaluronic acid (HA) [4], as well as polypeptide hydrogels such as gelatin and elastin [5]. Hydrogels can imbibe large amounts of water and, remarkably, swell to several times their dry weight [6], a behavior governed by the organization of water at the macromolecular interface of the hydrogel biomaterial [7]. Additionally, the molecular organization of water is dependent on the solute species, such as ions or molecules, present in the immediate environment.

Water molecules are composed of an electronegative oxygen atom that is flanked by electropositive hydrogen atoms on each side. These opposing charges facilitate hydrogen bonding, a secondary non-covalent interaction between water molecules, where the partial negative charge of the oxygen atom is shared with a hydrogen atom from another molecule. Water molecules can also interact via weak non-covalent bonding with other charged or polarized species such as solute ions or functional groups of a polymer. Therefore, the presence of ions or other molecules can impact hydrogen bonding between water molecules and influence the molecular organization of water molecules at the macromolecular interface.

In hydrogel systems, water molecules can be found in three different states: bound water, intermediate water, and free water. Each state is characterized by different mobility and abilities to crystalize [8]. Firstly, bound water molecules interact directly with polar functional groups at the polymer’s surface via hydrogen bonding, forming a layer of 1–4 molecules thickness (0.3–1.2 nm) [3], and thus have very little mobility. This water type is the first layer of hydration of hydrogels and is not crystallizable even at extremely low temperatures. Secondly, intermediate water is bound more loosely to polar groups at the polymer’s surface or to bound water molecules, forming the second hydration shell for hydrogel systems. The lower degree of interaction with the hydrogel polymer enables this water type to have higher mobility and to be crystallized at temperatures below 0 ºC. Lastly, free water presents characteristics close to bulk water, including high mobility and a crystallization peak around 0 °C because of its lack of direct interaction with the hydrogel polymer [8,9,10] (Figure S1). The three water states can also be categorized as non-freezable bound water (*W_nfb_*) and freezable water (*W_f_*), which includes the intermediate and free water. Studies investigating water states in natural and synthetic hydrogels, however limited, report clear correlations between the *W_nfb_*, the *W_f_*, and the equilibrium water content (*EWC*). Generally, the proportion of *W_f_* increases linearly with the *EWC*. The *W_nfb_* similarly increases with the *EWC* but reaches a plateau as it is limited by the available polymer surface area [10,11,12]. Therefore, the quantitative proportionality of different water states depends on the polymer type, particularly the polymer functional groups, their polarity, distribution along the polymer chain, and the polymer concentration. Several techniques can be applied to investigate the different water states of hydrogel systems. Such techniques include differential scanning calorimetry (DSC) [13,14,15,16,17], nuclear magnetic resonance spectroscopy (NMR) [18], X-ray diffraction [13], Fourier-transform IR spectroscopy (FTIR) [19], and terahertz spectroscopy [20].

Crosslinked gelatin methacryloyl (GelMA) is a versatile semi-synthetic hydrogel with highly tunable mechanical and diffusive properties [21], as well as low immunogenicity [22], that is well established for applications including tissue engineering, in vitro 3D cell culture models [23,24,25,26,27], and drug delivery [28,29,30,31,32]. GelMA is a derivative of gelatin, most commonly type A, which is functionalized with methacryloyl groups to facilitate photocrosslinking in the presence of a photoinitiator and light [24,25,33,34,35,36].

Current scientific literature for water dynamics in GelMA hydrogels is sparse and is limited to the study of the swelling behavior on a macroscopic scale, with the total water content as the main output [37,38,39,40,41]. To date, the molecular interaction and organization between GelMA and water have not been reported.

The swelling properties of hydrogels are directly related to the hydrogel crosslinking density and, therefore, the mesh size. These physical parameters also govern the diffusive properties of hydrogels, and therefore are critical in drug delivery [25,42]. As established, the swelling behavior and mesh size of hydrogels are highly sensitive to the surrounding environment, with temperature, pH, and ionic strength as influential factors [37,43,44]. Many hydrogel types, including GelMA, are being developed for human implantation, for example, in tissue engineering or drug delivery applications. Consequently, one has to consider the effects of microenvironmental factors of different in vivo environments. While the body temperature is reasonably stable, the pH and ionic strengths may vary between different tissues and organs. A classic example is the gastrointestinal tract, where pH and ionic strength vary greatly depending on the organ. The pH is acidic in the stomach (pH 2) and becomes basic in the intestine (pH 5 to 8) [45]. Blood, on the other hand, has a pH value of 7.4 [46]. The microenvironmental pH can also vary and change during pathogenesis and disease progression, and, for example, become acidic in an immediate tumor environment [47,48], during the wound healing process [49], or in the case of infection [46]. The ionic strength also varies depending on the organ and disease state. In the intestinal tract, it varies from close to 0 M in the stomach to 0.4 M in the intestine, in relation to a fed or fasted state [50,51,52,53,54]. The general physiological ionic strength is 150 mM [46].

Since polar or ionic atoms can form non-covalent bonds with water, hydrophilic drugs, when encapsulated in a hydrogel system for drug delivery purposes, may impact the molecular arrangement of water in the system. This is especially the case for small drugs that can easily diffuse throughout the hydrogel matrix and reach the first layer of hydration constituted by *W_nfb_*. Such molecules can interact with both water and the hydrogel, thereby potentially changing the proportional distribution of water states in the system [55].

In this work, we first characterized the crosslinking and swelling conditions of GelMA hydrogels and the impact of ionic strength on hydrogel swellability, as well as the water state distribution. Then, we performed a systematic study of the effect of a wide range of both ionic strength and pH of the hydrogel swelling media on the water state distribution, swellability, hydrogel mesh size, and the mechanical properties of GelMA hydrogels. Finally, we encapsulated a model drug, cefazolin, into GelMA hydrogels to investigate the potential impact of the negatively charged and polar antibiotic on the water state distribution.

## 2. Materials and Methods

### 2.1. Drug Encapsulation and GelMa Crosslinking

A mold casting technique was used to manufacture the GelMA hydrogels. Disc-shaped hydrogel samples of ~35 µL volume, measuring 5 mm diameter and 1.8 mm height, were made using polytetrafluoroethylene (PTFE) molds. Hydrogels were prepared by photocrosslinking 5%, 10% or 15% GelMA from gelatin A (Gelomics Pty Ltd., Brisbane, QLD, Australia) solution in ultrapure water (Milli-Q^®^, Merck Group, Darmstadt, Germany) or phosphate-buffered saline (PBS, Oxoid, Thermo Fischer Scientific, Waltham, MA, USA) in the presence of 0.5 mg/mL photoinitiator Irgacure 2959 (1-[4-(2-hydroxyethoxy)-phenyl]-2-hydroxy-2-methyl-1-propanone, BASF, Ludwigshafen, RLP, Germany). To study the effect of antibiotic loading, cefazolin (Sigma-Aldrich, St. Louis, MO, USA) was blend-loaded at final doses of 0, 3, 15, 30, or 90 µg per hydrogel sample, as indicated.

Ultraviolet (UV) crosslinking was applied for 30 min at 365 nm (intensity of ~2.6 mW/cm^2^ in a CL-1000 crosslinker; UVP, Upland, CA, USA). All GelMA concentrations are % *w/v* unless specified otherwise. Hydrogel samples were used for in vitro assays directly after manufacture.

### 2.2. Hydrogel Swelling

#### 2.2.1. Swelling Buffer Preparation

Hydrogel samples were swelled for 7 days at 37 °C in 1× PBS (Oxoid, Thermo Fischer Scientific) or ultrapure water (Milli-Q^®^, Merck Group, Darmstadt, Germany) for the first study focusing on the impact of the crosslinking and swelling media on water state. Then the ionic strength of the swelling media was assessed by using different PBS concentrations: 0.1×, 0.5×, 1×, 2×, respectively, corresponding to 15 mM, 75 mM, 150 mM, and 300 mM. The pH of the swelling media was evaluated by adjusting the pH of 0.5× PBS with aqueous NaOH and HCl to pH 2.5, 5, 7.4, 9, and 11, and the molarity of the adjusted swelling buffers was supplemented with NaCl to reach a fixed concentration of 150 mM that corresponds to the physiological value.

#### 2.2.2. Equilibrium Swelling

GelMA hydrogel samples (*n* = 8) were swelled in different media for 7 days to ensure that equilibrium was reached. The sample weight was recorded immediately after crosslinking/before swelling, and after 7 days of swelling. After the swelling, *n* = 5 samples were lyophilized using an ALPHA 1–4 LD_plus_/2–4 LD_plus_ unit (Martin Christ Gefriertrocknungsanlagen GmbH, Osterode, Germany) for 7 days to ensure complete lyophilization. The dry weight was used to calculate the equilibrium water content (*EWC*) using Equation (1):

(1) $$EWC\;\left(\%\right)=\frac{\left(m_{wet}-m_{lyophilized}\right)}{m_{wet}}\times 100$$

where $m_{Wet}$ is the wet mass at equilibrium swelling and $m_{lyophilized}$ is the mass after lyophilization of the hydrogel samples. The mass due to retention of salt in the hydrogels after lyopilization was not taken into account in these calculations for the presented data, because such a contribution was found to result in <1% difference in the calculated values when analyzed for a representative spread of samples across all experimental conditions.

The remaining *n* = 3 samples were used for DSC analysis to determine the different water states. Hydrogels loaded with the cefazolin drug were not swelled in order to prevent drug loss from the hydrogel but were analyzed by DSC immediately upon crosslinking.

#### 2.2.3. Equilibrium Mass Swelling Ratio

Samples were immersed in swelling media (PBS or water, as indicated) at 37 °C for 7 days, until constant mass to ensure that equilibrium was reached. Samples (*n* = 8) were weighed after the 7-day swelling and then after lyophilizing (*n* = 5). The swelling media was refreshed each day. The equilibrium mass swelling ratio $Q_{m}$ was calculated according to Equation (2) where $m_{wet}$ is the mass of the hydrogel samples after swelling and $m_{lyophilized}$ is the dry mass after lyophilizing of the hydrogels:

(2) $$Mass\;swelling\;ratio=Q_{m}=\frac{\left(m_{wet}-m_{lyoplilized}\right)}{m_{lyophilized}}$$

#### 2.2.4. Mesh Size Calculation

The mesh size ξ of GelMA hydrogels was determined using the mass swelling ratio obtained experimentally, the volume fraction, and the gelatin and GelMA hydrogel characteristics [56,57] in a four-step calculation.

First, the relaxed mass swelling ratio $Q_{mr}$ and the equilibrium mass swelling ratio $Q_{m}$ were obtained with Equation (2) using the wet weight immediately after crosslinking and after reaching equilibrium swelling, respectively. $Q_{mr}$ and $Q_{m}$ were then used to calculate the relaxed volumetric swelling $Q_{vr}$ and the equilibrium volumetric swelling $Q_{v}$, respectively, using Equation (3), where the PBS density was used as solvent density ($\rho_{s}=1.014\;g/{\mathrm{cm}}^{3}$) [58] and the gelatin density as polymer density ($\rho_{p}=1.35\;g/{\mathrm{cm}}^{3}$) [59,60,61].

(3) $$Q_{v\left(r\right)}=1+\frac{\rho_{p}}{\rho_{s}}\left(Q_{m\left(r\right)}-1\right)$$

Secondly, the relaxed polymer volume fraction $v_{2r}$ and the equilibrium polymer volume fraction $v_{2s}$ were calculated using Equation (4).

(4) $$v=\frac{1}{Q_{v}}$$

Thirdly, the Flory–Rehner Equation (5), used for polymers crosslinked in solvents, [56,62,63] was used to calculate the molecular weight between crosslinks $M_{c}$ (g/mol). The following polymer properties were used for the calculation: the specific volume of the polymer $\bar{v}$ = 0.7407 mL/g [64]; the molar volume of the solvent $V_{1}$ = 18.01 mL/mol for water; the polymer–solvent interaction $X_{1}$ = 0.497 (also known as the Flory’s Chi parameter) [65]; and the number average molecular weight before crosslinking $M_{n}$ = 63,565 g/mol [66].

(5) $$\frac{1}{M_{c}}=\frac{2}{M_{n}}-\frac{\frac{\bar{v}}{V_{1}}\;[\ln\left(1-v_{2s}\right)+v_{2s}+X_{1}v_{2s}{}^{2}]}{v_{2r}\left[{\left(\frac{v_{2s}}{v_{2r}}\right)}^{\frac{1}{3}}-\frac{1}{2}\left(\frac{v_{2s}}{v_{2r}}\right)\right]}\;$$

Finally, the mesh size ξ (nm) was calculated using Equation (6). $M_{r}$ = 91.19, the molecular weight of the repeat unit, was taken as the average molecular weight of the amino acid composition [64]. The amino acid bond length $l=4.28\;\dot{A}$ [67], and the Flory’s characteristic ratio for GelMA $C_{n}$ = 8.8785 [64] were used.

(6) $$\xi =v_{2s}{}^{-\frac{1}{3}}\times l\;{\left(2\;\frac{M_{c}}{M_{r}}C_{n}\right)}^{\frac{1}{2}}$$

### 2.3. Differential Scanning Calorimetry

A NETZCH differential scanning calorimeter (DSC) 204 F1 Phoenix^®^ was used to apply a cooling/heating cycle under nitrogen flow. Swelled hydrogels were cut and weighed to obtain samples (*n* = 3) of 5–8 mg that were individually sealed in a T-zero hermetic pan. Cooling from 20 °C (room temperature) to −40 °C followed by heating from −40 °C to 90 °C was performed at a rate of 10 °C per minute. The enthalpy of the water melting peak was obtained and used to determine the water states according to Equations (7) and (8) [8]:

(7) $$W_{f}\;\left(\%\right)=\frac{\Delta_{m}}{\Delta H_{m}}\times 100$$

where $W_{f}$ is the freezable water fraction in weight percentage, $\Delta_{m}$ is the enthalpy of the water melting peak obtained from the DSC thermograms expressed in J/g, and $\Delta H_{m}$ is the enthalpy of bulk water (334 J/g) [68].

(8) $$W_{nfb}\;\left(\%\right)=EWC-W_{f}$$

The non-freezable bound water $W_{nfb}$, expressed in weight percentage, is obtained by subtracting the freezable water $W_{f}$ from the equilibrium water content EWC, both expressed in weight percentage.

### 2.4. Mechanical Compression Test

GelMA hydrogel samples (*n* = 8) were immersed in the appropriate swelling media in an unconfined compression test after the 7-day swelling. An Instron 5848 microtester with a 500 N load cell (Instron, Melbourne, VIC, Australia) was used to apply unconfined compression with a displacement rate of 0.01 mm/s using a non-porous aluminum indenter. The Young’s compressive modulus was determined from the slope of the stress-strain curve, between 10% and 15% strain. The failure stress and strain were defined as the coordinates of the maxima of the stress-strain curve before the hydrogel sample cracked (sudden drop in the stress-strain curve).

### 2.5. Statistical Analysis

Probabilities of *p* ≤ 0.05 were considered significant differences. The significance of mean differences between groups was calculated using the general linear model (univariate analysis), using IBM SPSS Statistics 23 (IBM Corp). Ns = non-significant; * = *p* < 0.05; ** = *p* < 0.01; *** = *p* < 0.001; **** = *p* < 0.0001.

## 3. Results and Discussion

### 3.1. Impact of the Crosslinking and Swelling Media on Water State

Ions naturally disrupt the organization of surrounding bulk water molecules by recruiting them to form a hydration shell around themselves [69]. Therefore, we hypothesized that the presence of ions would affect the quantitative distribution of water states in GelMA hydrogels. Therefore, we investigated the effects of ionic strength and polymer weight fraction on the water state distribution and molecular arrangement of water within GelMA hydrogel constructs. GelMA was dissolved in water or PBS, respectively, at concentrations ranging from 5% to 15%, and subsequently allowed to swell in water or PBS for 7 days.

**Figure 1:**
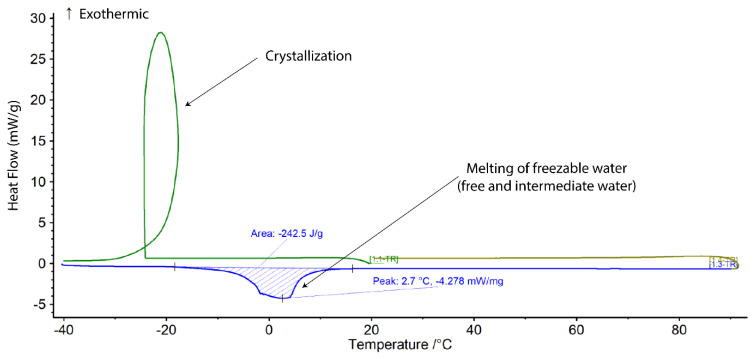
Differential scanning calorimetry (DSC) thermogram of 15% GelMA crosslinked in PBS and swelled in PBS.

Figure 1 shows a representative DSC thermogram of both the heating scan (shown in blue) and cooling scan (shown in green) for 15% GelMA crosslinked in PBS and swelled in PBS. The crystallization of free and intermediate water appears together as a ‘crystallization loop’. The loop is an artefact, due to the large exotherm of water crystallization. The freezable water fraction (*W_f_*) was calculated according to Equation (7) and subsequently non-freezable bound water (*W_nfb_*) could be calculated according to Equation (8).

**Figure 2:**
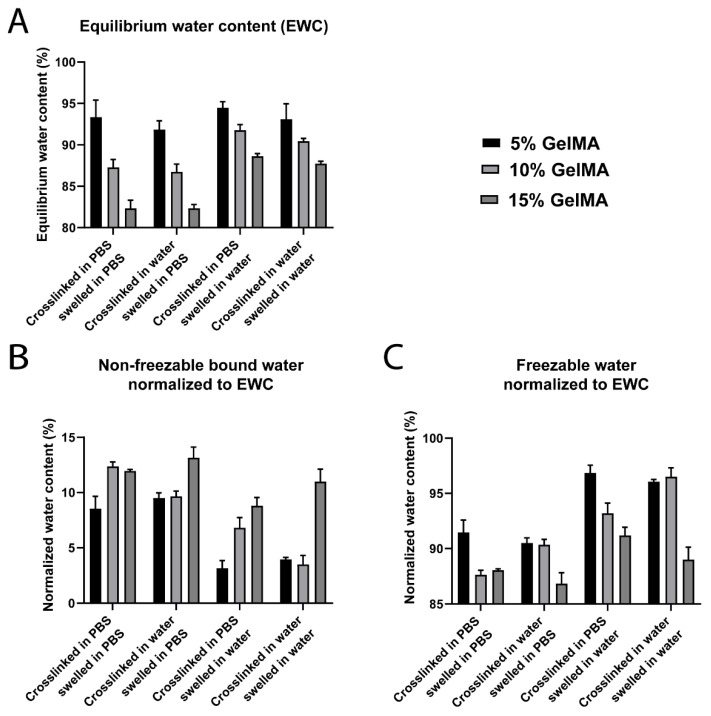
Water content and water types for GelMA hydrogels (5% to 15% gel fraction) crosslinked and swelled in different media. (**A**) Equilibrium water content (*EWC*, *n* = 5). (**B**) Non-freezable bound water (*W_nfb_*) normalized to the *EWC* (*n* = 3). (**C**) Freezable water (*W_f_*) normalized to the *EWC* (*n* = 3).

GelMA hydrogels showed great sensitivity to the ionic strength of the surrounding medium (Figure S2 and Figure 2). Regardless of the crosslinking and swelling media conditions, the fraction of *W_nfb_* increased with GelMA concentration. This trend was expected because the number of polar sites for hydrogen bonding, as well as the surface area for interaction between the polymer and the water molecules, increases with GelMA concentration [12]. This phenomenon has previously been described by Ostrowska-Czubenko et al., as well as Rodríguez-Rodríguez et al., who collectively demonstrated an increase of *W_nfb_*, associated with an increase of polymer surface area and functional groups, that eventually reached a plateau once all polar groups were saturated [10,11]. Additionally, the *W_nfb_* fraction tended to be higher in hydrogels swelled in PBS (Figure S2A,B) than those swelled in water (Figure S2C,D).

The equilibrium water content (*EWC*) is directly related to the swelling behavior of hydrogels and describes the proportion of water when the equilibrium between the mechanical tension of the hydrogel network and the osmotic pressure of the surrounding media is reached. Osmotic pressure can be divided into the water osmotic pressure and the ion osmotic pressure, with both water molecules and ions diffusing in and out of the hydrogel network to different degrees based on their respective gradients [70]. Higher GelMA concentration presented lower *EWC* due to higher crosslinking density and, hence, higher elastic forces opposing the swelling [70] (Figure 2A). Notably, the crosslinking media had no significant impact on the *EWC*. GelMA hydrogels swelled in PBS displayed lower *EWC* than those swelled in water (Figure 2A). While hydrogels swelled in PBS experienced both an inward (due to the presence of the polymer network) and outward (due to higher ion concentration in the media) water gradient, hydrogels swelled in water experienced only an inward water gradient, and hence displayed the highest *EWC* and associated swelling (Figure 2A and Figure S3C, respectively).

Non-freezable bound water data normalized to the *EWC* confirmed the overall trend of increasing *W_nfb_* when the GelMA concentration increased (Figure 2B). Hydrogels swelled in PBS showed higher *W_nfb_* content compared to those swelled in water. Conversely, the *W_f_* content was higher for hydrogels swelled in water compared to those swelled in PBS (Figure 2C). The results suggest that the presence of ions in the swelling media enhanced the non-freezable bound water fraction in the GelMA hydrogels.

**Figure 3:**
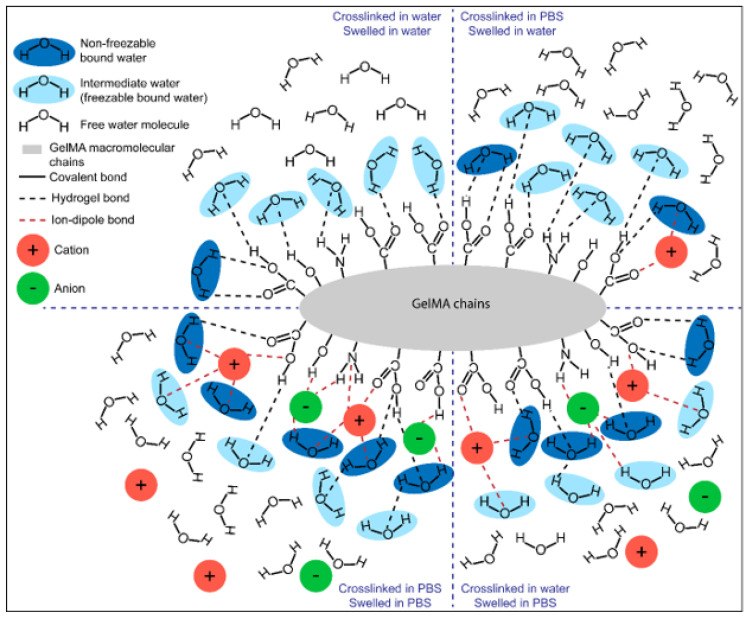
Graphic illustration of the water content in GelMA hydrogels under different crosslinking and swelling conditions. *W_f_*, characterized by intermediate mobility and crystallizability, are represented in light blue and with one hydrogen bond. *W_nfb_* molecules in dark blue have two bonds (hydrogen bond or ion–dipole bond), have the lowest mobility, and are not crystallizable. In the top-left, no ions are present; therefore, the *W_nfb_* is minimal. In the top-right, the introduction of ions from the crosslinking media leads to the formation of ion–dipole bonds between ion, water, and polymer functional groups, thereby reinforcing the *W_nfb_* fraction, yet with minimal effect on swelling since the swelling media does not contain ions. When the swelling media is PBS, (lower-left and right), the higher ion concentration reinforces the *W_nfb_* fraction via ion–dipole bonds between GelMA functional groups and water.

Figure 3 schematically recapitulates the proposed polymer–water interaction in the different groups tested for this study. We attribute the increase of *W_nfb_* in GelMA hydrogels swelled in PBS to the presence of strong ion–dipole bonds with both water molecules and the polar/charged hydrogel functional groups. Ions appear to play a key role in recruiting *W_nfb_* molecules and the formation of the first layer of hydration. For GelMA hydrogels swelled in water, the absence of ions or the minimal level of ions in hydrogels crosslinked in PBS, the ion–dipole bonds are essentially absent, and hydrogen bonds are the primary interaction between water and the hydrogel. This hypothesis also seems to be valid for neutral hydrogels, particularly poly (ethylene glycol) diacrylate hydrogels, for which water state distributions were affected similarly by ionic strength [71].

### 3.2. Impact of the Ionic Strength of the Swelling Media

It is well known that polyelectrolyte hydrogels are highly sensitive to environmental factors such as temperature, pH, and ionic strength [37,43,44]. In light of the results of the first section, pointing at the significantly greater influence of the swelling media composition (PBS or water) compared to the crosslinking media, for subsequent sections of the study, we chose to crosslink GelMA hydrogels in water to investigate the effects of ionic strength of the swelling media, and to ensure that no additional ions were introduced into the GelMA systems at the crosslinking step.

**Figure 4:**
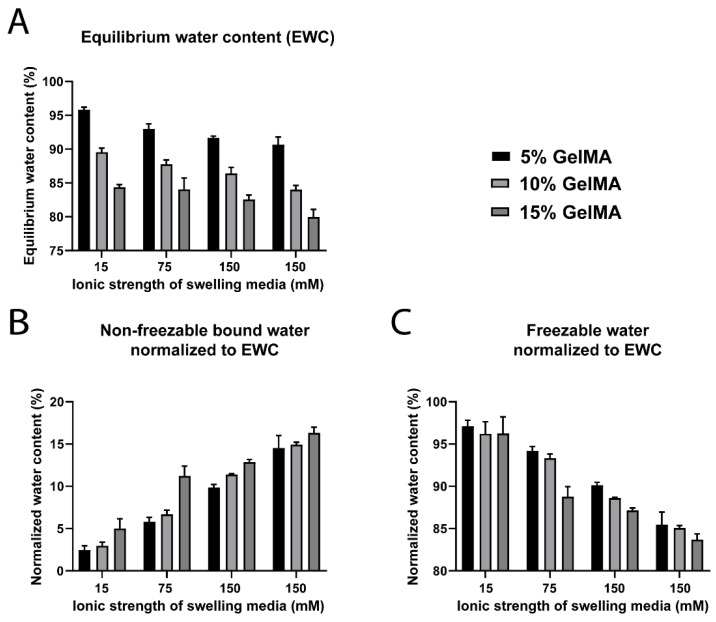
Water content and water types for GelMA hydrogels (5% to 15% gel fraction) crosslinked in water and swelled at different ionic strengths. (**A**) Equilibrium water content (*EWC*, *n* = 5). (**B**) Non-freezable bound water (*W_nfb_*) normalized to the *EWC* (*n* = 3). (**C**) Freezable water (*W_f_*) normalized to the *EWC* (*n* = 3).

GelMA hydrogels presented a gradual impact of the swelling media ionic strength on the water content and distribution (Figure 4, Figures S4 and S5). As expected, the *EWC* decreased with increasing GelMA concentration (Figure 4A and Figure S4). The *W_nfb_* increased with GelMA concentration and ionic strengths 15 mM up to and including 150 mM, confirming the trend observed in Section 3.1. There was no significant increase in *W_nfb_* from 5% to 15% GelMA swelled in 300 mM media, suggesting that the polymer polar functional groups were saturated (Figure S4D). The increase in ionic strength of the swelling media created an increasing outward osmotic water pressure from the hydrogels, thereby decreasing the *EWC* (Figure 4A). The *W_nfb_* content normalized to the *EWC* significantly increased from ~2.5 to ~16.3% with ionic strength, likely due to reinforcement of the tightly bound first hydration layer by ion–dipole interactions, as hypothesized in Section 3.1. An additional effect that is expected to come into play is ionic bridging between polymer chains. At physiological pH, the deprotonated carboxyl groups can form ionic crosslinks, particularly with divalent ions such as Ca^2+^ or Mg^2+^, subsequently modifying swelling and mechanical properties of the hydrogels. This effect is well documented for polyelectrolyte hydrogels, such as alginate [72]. The presence of such ionic crosslinks acts to further decrease *EWC*, in addition to the other effects already described. However, in the present GelMA system this would be expected to be a relatively minor contributor to the overall properties, since the majority of the positively charged ions in PBS are monovalent (Na^+^). The *W_f_* content decreased from ~97 to ~83.5% (Figure 4B,C), due to the overall lower water content of these gels. These results confirmed the importance of ionic strength in the proportional distribution of water states in GelMA hydrogels and demonstrate that the presence of ions favors an increase of the *W_nfb_* fraction.

**Figure 5:**
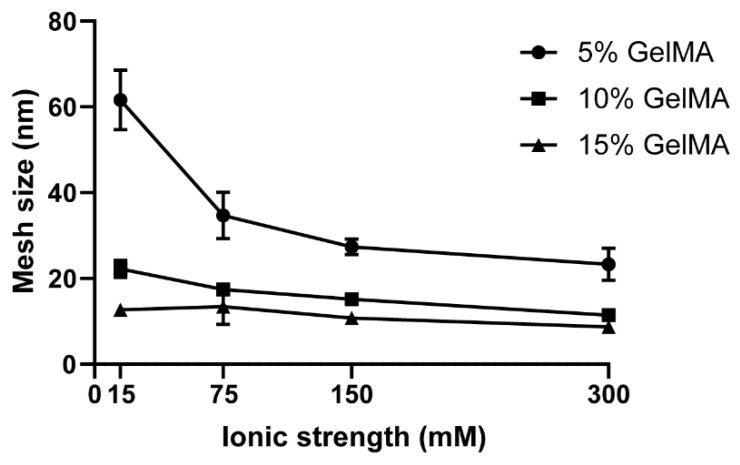
Mesh size for GelMA hydrogels (5% to 15%) crosslinked in water and swelled at different ionic strengths.

The hydrogel mesh size is derived from the equilibrium swelling ratio (See Materials and Methods section and Figure 5 and Figure S6) and was most significantly impacted by the ionic strength of the swelling media at low GelMA concentrations of 5%, compared to 10% and 15%, but overall followed a decreasing trend with increasing ionic strength (Figure 5). These were expected results and have previously been reported for hydrogels in other studies [64,73]. Across all GelMA concentrations, the mesh size varied from 23 to 4 and 61.6 to 8.7 nm.

**Figure 6:**
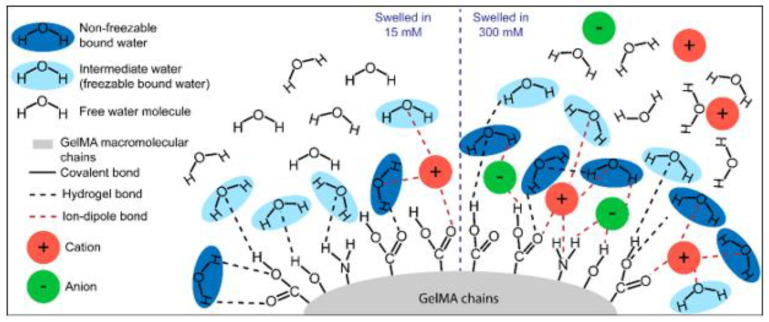
Graphic illustration of the proposed mechanism of water–GelMA interactions in GelMA hydrogels swelled at different ionic strengths. *W_f_*, *W_nfb_* and free water molecules are presented with the same conventions as in Figure 3. On the right, the highest ionic strength of the swelling media reinforces the *W_nfb_* fraction, more than for the lowest ion concentration on the left, due to increased number of ion–dipole bonds.

Higher mesh size may be expected to correlate with lower mechanical stiffness due to the higher swelling ratio of these samples. Therefore, the mechanical properties of the hydrogels were evaluated to elucidate the potential impact of the ionic strength of the swelling media on the compressive modulus, failure stress, and failure strain for GelMA hydrogels (Figures S7 and S8). The failure strain, failure stress and compressive modulus were largely unaffected by the ionic strength of the swelling media (see Figure S7) and agrees with our previous work [74]. A potential mechanism of molecular interaction of GelMA with water is illustrated in Figure 6 for the lowest and highest ionic strengths (15 nM and 300 nM) investigated here. The gradual increase in ion concentration in the swelling media favors the recruitment of *W_nfb_* ion–dipole bonds between the ions and water molecules and/or GelMA functional groups. This section’s results confirmed the hypothesis elaborated in Section 3.1 and also reported by Yang et al. [71].

The water content of hydrogels, particularly at the polymer interface, plays an essential role in hemocompatibility [8], as well as protein adsorption and folding [2]. The literature demonstrates that higher intermediate water levels may be associated with low platelet adhesion, which is correlated with greater physiological hemocompatibility [8]. Here, the notion of hemocompatibility means that the polymer does not cause aggregation of blood cells or adsorb proteins, which could trigger the body’s immune response [2]. We would argue that in the framework of tissue engineering strategies, the ability to adsorb proteins and adhere platelets on the polymer surface favors the tissue healing process, and therefore its regeneration. Although a strong inflammation is not desirable, a lower inflammatory response is needed to initiate the tissue healing process. The chronology of events after implanting medical devices corresponds to the wound healing process [75]. First, coagulation occurs with a high platelet level on-site. Inflammation then follows, with the recruitment of immune cells such as neutrophils and macrophages. These events are a necessary cascade that ends with the proliferation of fibroblasts to remodel the tissue [75]. Because intermediate water is included in the *W_f_* water state [8], we can hypothesize that, in contrast, higher levels of *W_nfb_* would tend to promote platelet, cell, or protein adhesion, thereby promoting wound healing and tissue regeneration. Our results, therefore, suggest that higher ionic strength media would be preferred in a GelMA system to promote tissue regeneration.

### 3.3. Impact of the pH of the Swelling Media

For peptide-based hydrogels, the pH of the surroundings almost always influences the properties of the construct and particularly the swelling capabilities of the hydrogels [44,76]. Therefore, we evaluated the water content and states of 5%, 10%, and 15% GelMA hydrogels swelled in PBS at a fixed ionic strength (150 mM) but varying pH values, ranging from 2.5 to 11.

**Figure 7:**
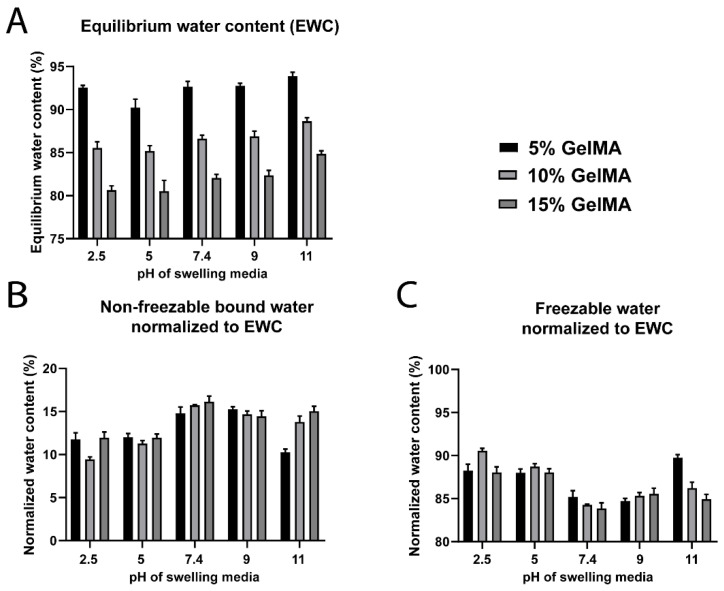
Water content and water types for GelMA hydrogels (5% to 15% gel fraction) crosslinked in water and swelled in media of different pH and fixed ionic strength of 150 mM. (**A**) Equilibrium water content (*EWC*, *n* = 5). (**B**) Non-freezable bound water (*W_nfb_*) normalized to the *EWC* (*n* = 3). (**C**) Freezable water (*W_f_*) normalized to the *EWC* (*n* = 3).

Absolute *W_nfb_* was significantly higher under neutral and basic swelling conditions compared to lower pH values (Figures S9C–E and S10A). The same trend was confirmed in normalized datasets (Figure 7), which demonstrated that the *W_nfb_* levels were higher at pH 7.4, 9, and 11 (~15%), while the level was ~12.5% for the acidic pH values (Figure 7B). Conversely, the *W_f_* was ~88–90% for pH 2.5 and 5, and ~85% for pH 7.4 to 11 (Figure 7C). The *EWC* was similarly impacted (Figure 7A), but an inflection point at pH 5 was noted and more pronounced for 5% GelMA hydrogels. Hollingshead et al. showed that their peptide-based, pH-sensitive hydrogel presented lower water content at pH close to the isoelectric point of the peptide and significantly increased at pH 11 [77]. At a pH close to its isoelectric point, their peptide-based hydrogel, as expected, presented a lower water content. On the contrary, at pH 11, above the isoelectric point, the hydrogel was highly negatively charged, and thus presented a significant increase in water content [77]. Here the inflection point for GelMA hydrogel water content was at pH 5, which is lower than the reported isoelectric point of Gelatin A (pH 7–9) [78,79,80]. This may be related to a shift of the isoelectric point associated with the methacrylation of primary amines. GelMA hydrogels also presented the highest *EWC* at pH 11 (Figure 7A), which is consistent with the findings of Hollingshead et al. [77].

**Figure 8:**
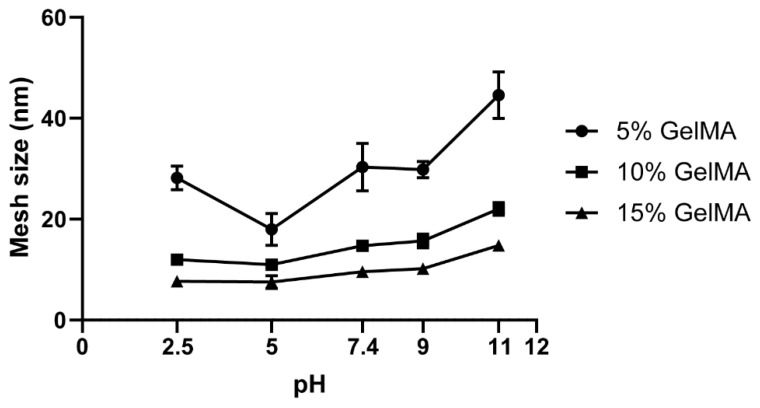
Mesh size for GelMA hydrogels (5% to 15%) crosslinked in water and swelled at different pH.

The mesh size of the hydrogels, derived from the equilibrium swelling ratio (Figure S11), presented a slight and gradual increase from pH 2.5 to 11 (Figure 8). The 5% GelMA groups showed higher sensitivity to the pH variation and an inflection point at pH 5, correlating with the *EWC* results (Figure 7A) and previous studies [77].

**Figure 9:**
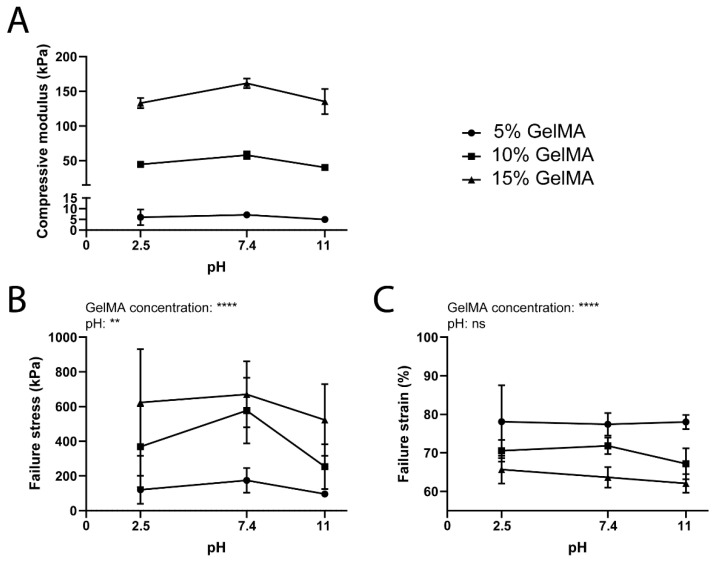
Mechanical properties for GelMA hydrogels (5% to 15% gel fraction) swelled at different pH. (**A**) Compressive modulus. (**B**) Failure stress. (**C**) Failure strain. Data are shown as means ± standard deviation, *n* = 6–8. ** = *p* < 0.01; **** = *p* < 0.0001.

The mechanical properties of GelMA hydrogels were more sensitive to changes in the pH than the ionic strength of the swelling media (Figure 9 and Figure S12). In particular, the compressive modulus was highest at pH 7.4 for all GelMA concentrations compared to both basic and acidic conditions (Figure 9A). Although the error bars are large in Figure 9B,C, and consequently no definitive statements can be made regarding this data, the failure stress also showed an apparent incline at pH 7.4, while the failure strain was not significantly affected by the pH of the swelling media. Since the isoelectric point of gelatin A is around pH 7–9 [78,79,80], our results agree with Hollingshead et al., who also demonstrated that peptide hydrogels were significantly stiffer at a pH close to the isoelectric point [77]. Overall, the Young’s modulus and the failure stress increased from 5 kPa to 161 kPa and 96 kPa to 670 kPa, respectively, with an increasing GelMA concentration. On the contrary, the failure strain decreased from 78% to 62% for increasing GelMA concentration. The variation of the three parameters according to the GelMA concentration is in line with our previous findings [74].

**Figure 10:**
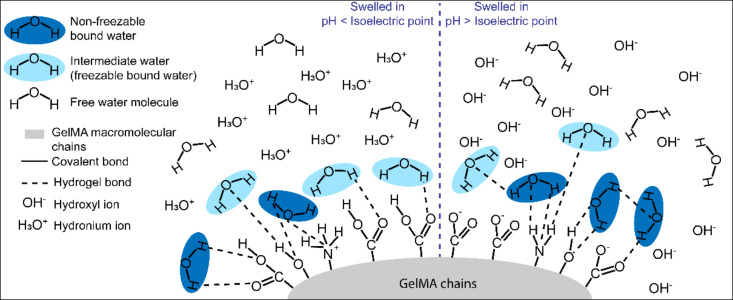
Graphic illustration of the water content in GelMA hydrogels swelled at different pH. *W_f_*, *W_nfb_* and free water molecules are presented with the same conventions as in Figure 3. On the left, when the pH of the swelling media is below the GelMA isoelectric point, the amine functional groups are protonated, thus providing an overall positive charge at the surface of GelMA. Because the amine groups are in the minority compared to the carboxylic acid groups, the charge and effect on the water content and states is minimal. On the right, when the pH of the swelling media is above the GelMA isoelectric point, the carboxylic functional groups tend to be deprotonated, giving an overall negative charge to the hydrogel, making it more hydrophilic and thus imparting a higher water content and higher *W_nfb_*.

A schematic representation of GelMA hydrogel functional groups at pH below and above the hydrogel’s isoelectric point are presented in Figure 10. The amino acid composition of type A gelatin can vary depending on the animal source, but it is notably composed of: serine, threonine, hydroxyproline, and hydroxylysine residues which present a hydroxyl group on their side-chain, as well as lysine and hydroxylysine residues that provide an amine side chain group; and aspartic acid and glutamic acid residues with a carboxylic acid side chain group [35]. While the lysine and hydroxylysine typically represent 5% of the gelatin amino acid composition, the glutamic and aspartic acid are three times more highly represented in gelatin (16%) [35]. Also, during the methacrylation reaction of gelatin, a proportion of the amine and hydroxyl groups are functionalized, with the amine groups reacting preferentially [33,78,81]. As a result, the proportion of free amine side groups is decreased by around 80% (corresponding to the degree of functionalization). After crosslinking, the free carboxylic acid functional groups are predominant compared to the amine groups.

When a GelMA hydrogel is allowed to swell in media with a pH that is higher than its isoelectric point, the carboxyl groups of the amino acid residues are proportionally more deprotonated than protonated and, as a result, the biopolymer presents an overall negative charge. Conversely, at acidic pH below the isoelectric point, the amine groups are protonated, presenting an overall positively charged polymer. Still, since the concentration of amine groups is lower than the carboxylic acid groups [35], there is a more potent effect of the pH above the isoelectric point. The protonated amine groups and deprotonated carboxylic acid groups do not form hydrogen bonds with water molecules because they are involved in an acid–base reaction with water. Therefore, they are not likely to be the causative factor for the variation in *W_nfb_*. It was demonstrated that, as opposed to neutral hydrogels, highly charged hydrogels are more hydrophilic, thus swell more, i.e., incorporate higher water content [77,82,83]. Consequently, we hypothesize that at higher pH, the negative charges of the carboxylic acid groups create electrostatic repulsion between the polymer chains, which consequently are further apart and promote a greater water intake and *W_nfb_* [84].

### 3.4. Impact of Drug Loading

**Figure 11:**
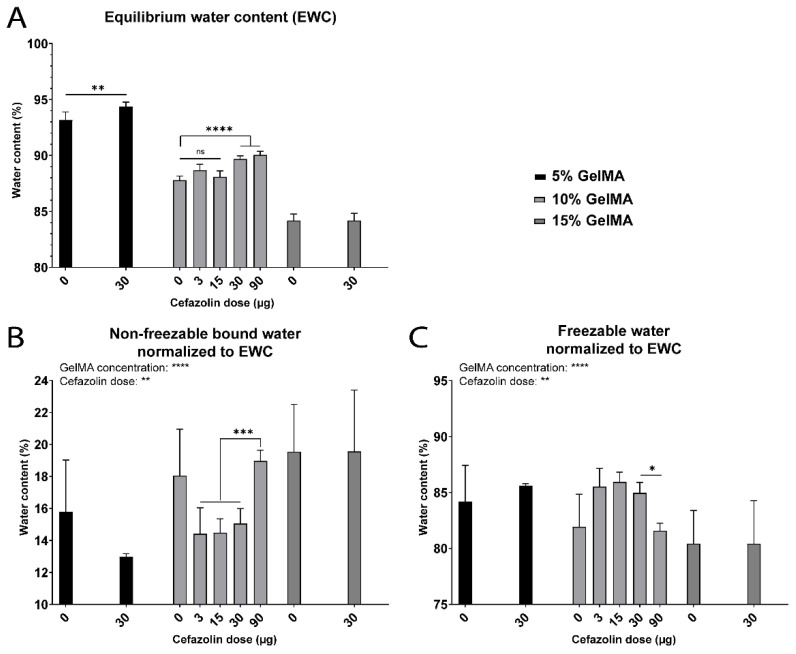
Water content and water types for GelMA hydrogels (5% to 15% gel fraction) crosslinked with 0, 3, 15, 30 or 90 µg cefazolin in PBS. Hydrogels were analyzed just after crosslinking. (**A**) Equilibrium water content (*EWC*) (%) (*n* = 5). (**B**) Non-freezable bound water (*W_nfb_*) (%) normalized to the *EWC* (*n* = 3). (**C**) Freezable water (*W_f_*) (%) normalized to the *EWC* (*n* = 3). Ns = non-significant; * = *p* < 0.05; ** = *p* < 0.01; *** = *p* < 0.001; **** = *p* < 0.0001.

The impact of encapsulation of the hydrophilic, ionic, and polar antibiotic cefazolin sodium on the water states in GelMA hydrogels was evaluated. Figure S13 shows the water content and state distribution of all the groups tested. While the presence of cefazolin did not impact the *W_nfb_* content for 5% and 15% GelMA, the obtained results showed a significant decrease in *W_nfb_* for 10% GelMA containing 3, 15, or 30 µg cefazolin. In contrast, the highest cefazolin dose of 90 µg encapsulated in 10% GelMA displayed a *W_nfb_* content similar to the control group, 10% GelMA. This represents an increase in *W_nfb_* for the 90 µg dose compared to the other cefazolin doses, suggesting a potential effect of the cefazolin dose (Figure 11B). The *EWC* inversely correlated with the GelMA concentration but increased with the presence of cefazolin in a dose-dependent manner for 5% and 10% GelMA and high doses of cefazolin (30 µg and 90 µg) (Figure S13 and Figure 11A).

**Figure 12:**
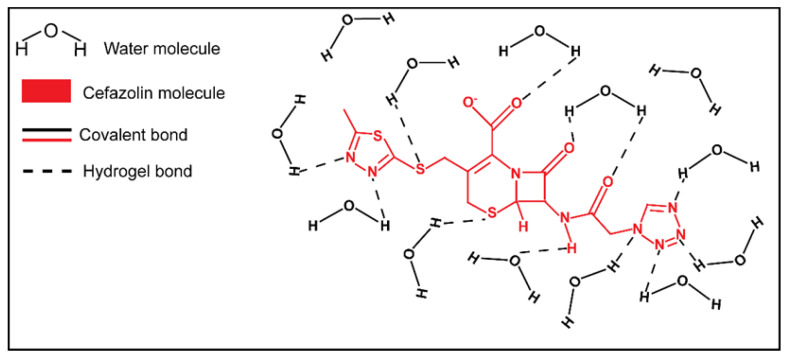
Graphic illustration cefazolin interaction with water molecules at pH 7.4. The polar hydrogen, nitrogen, sulfur, and oxygen atoms can form hydrogen bonds with water molecules.

While the absence of cefazolin dose effect for 5% and 15% GelMA groups could not be explained at the drug doses tested, the increase of *W_nfb_* in 10% GelMA presented for the highest cefazolin dose compared to the lower doses could be explained by the chemical structure of cefazolin (Figure 12). Cefazolin contains several electronegative nitrogen, sulfur and oxygen atoms, and electropositive hydrogen atoms [85]. Both types of atom can interact and bind water molecules via hydrogen bonds. Cefazolin is also negatively charged at physiological pH due to deprotonation of the carboxyl group and a pKa at 2.3 [85,86]. Consequently, cefazolin can potentially recruit water molecules to participate in strong ion–dipole bonding and subsequently reinforce the *W_nfb_* in the first layer of GelMA hydration, as discussed in earlier sections of this manuscript for ion–water interactions.

## 4. Conclusions

Our study confirmed a greater impact of the swelling media over the crosslinking media on the molecular organization of water in GelMA hydrogels. The systematic evaluation of the impact of the ionic strength confirmed an increase of *W_nfb_* with ionic strength. We propose a model of ion–dipole bonding between the ions of the swelling media and both the water molecules and the polymer functional groups. In this way, the ion–dipole bonds enhance the formation of bound water molecules, reinforcing the first layer of hydration of the polymer chains. The mechanism underlying the increase of *W_nfb_* at basic pH is likely related to electrostatic repulsion of the deprotonated carboxylic groups, thereby increasing the mesh size, swelling capacity, and overall intake of water. The observation that the presence of cefazolin in the GelMA hydrogels caused a decrease in the *W_nfb_* could not be fully explained by our study and requires further investigation. However, the highest cefazolin dose led to an increase in the *W_nfb_*, and we hypothesize that this is due to the drug’s molecular structure, which presents numerous polar atoms and a negatively charged atom that can form hydrogen bonds and ion–dipole bonds, respectively, with water. Overall, the ionic strength and pH impacted the mesh size (the primary physical parameter controlling drug release) [87], but also modified the molecular arrangement of water molecules at the polymer interface, as demonstrated by the *W_nfb_* and *W_f_* results. Since the hydration state and the molecular water layers at the surface of a polymer are thought to play a critical role in cell, protein, and platelet adhesion, water is expected to play a primary role in the interaction of polymers with biological systems [2], and therefore in tissue engineering applications [75]. Consequently, we deem the investigation of molecular water distribution to be of utmost importance in order to better understand the role of water in the potential application of hydrogels for biomedical use.

## Data Availability

Data is contained within the article and supplementary information.

## Supplementary Materials

The following are available online at https://www.mdpi.com/article/10.3390/biomedicines9050574/s1, Figure S1: The different water states in hydrated GelMA hydrogels, Figure S2: Water content for GelMA hydrogels (5 to 15%) crosslinked and swelled in different media, Figure S3: Wet weight, dry weight and equilibrium swelling ratio for GelMA hydrogels crosslinked and swelled in different media, Figure S4: Water content for GelMA hydrogels (5 to 15% gel fraction) swelled in media with increasing ionic strength, Figure S5: Wet weight and dry weight for GelMA hydrogels swelled in different ionic strengths, Figure S6: Equilibrium swelling ratio for GelMA hydrogels (5 to 15%) crosslinked in water and swelled at different ionic strengths, Figure S7: Mechanical properties for GelMA hydrogels (5 to 15% gel fraction) swelled at different ionic strength, Figure S8: Repesentative stress-strain curves for (A) 5%, (B) 10%, and (C) 15% GelMA hydrogels crosslinked in water and swelled at different ionic strengths, Figure S9: Water content for GelMA hydrogels (5 to 15% gel fraction) crosslinked in water and swelled in PBS at different pH, Figure S10: Wet weight and dry weight for GelMA hydrogels swelled in different pH, Figure S11: Equilibrium swelling ratio for GelMA hydrogels (5 to 15%) crosslinked in water and swelled at different pH, Figure S12: Repesentative stress-strain curves for (A) 5%, (B) 10%, and (C) 15% GelMA hydrogels crosslinked in water and swelled at different pH, Figure S13: Water content for GelMA hydrogels (5 to 15% gel fraction) crosslinked with 0, 3, 15, 30, or 90 µg cefazolin in PBS.

## References

[1] Caló E., Khutoryanskiy V.V. (2015). Biomedical applications of hydrogels: A review of patents and commercial products. Eur. Polym. J..

[2] Tanaka M., Hayashi T., Morita S. (2013). The roles of water molecules at the biointerface of medical polymers. Polym. J..

[3] Ratner B.D., Latour R.A. (2020). Role of Water in Biomaterials. Biomaterials Science.

[4] Pan H.M., Yu H., Guigas G., Fery A., Weiss M., Patzel V., Trau D. (2017). Engineering and Design of Polymeric Shells: Inwards Interweaving Polymers as Multilayer Nanofilm, Immobilization Matrix, or Chromatography Resins. ACS Appl. Mater. Interfaces.

[5] Kripotou S., Zafeiris K., Culebras-Martínez M., Gallego Ferrer G., Kyritsis A. (2019). Dynamics of hydration water in gelatin and hyaluronic acid hydrogels. Eur. Phys. J. E.

[6] Chai Q., Jiao Y., Yu X. (2017). Hydrogels for Biomedical Applications: Their Characteristics and the Mechanisms behind Them. Gels.

[7] Yang P., Mather P.T. (2020). Thermal Analysis to Determine Various Forms of Water Present in Hydrogels.

[8] Bag M.A., Valenzuela L.M. (2017). Impact of the hydration states of polymers on their hemocompatibility for medical applications: A review. Int. J. Mol. Sci..

[9] Higuchi A., Iijima T. (1985). D.s.c. investigation of the states of water in poly(vinyl alcohol-co-itaconic acid) membranes. Polymer.

[10] Ostrowska-Czubenko J., Pieróg M., Gierszewska-Drużyńska M. (2011). State of Water in Noncrosslinked and Crosslinked Hydrogel Chitosan Membranes–Dsc Studies. http://psjd.icm.edu.pl/psjd/element/bwmeta1.element.psjd-cb26791c-a142-4a1f-b035-93a7b390803d.

[11] Rodríguez-Rodríguez R., García-Carvajal Z.Y., Jiménez-Palomar I., Jiménez-Avalos J.A., Espinosa-Andrews H. (2019). Development of gelatin/chitosan/PVA hydrogels: Thermal stability, water state, viscoelasticity, and cytotoxicity assays. J. Appl. Polym. Sci..

[12] Ping Z.H., Nguyen Q.T., Chen S.M., Zhou J.Q., Ding Y.D. (2001). States of water in different hydrophilic polymers-DSC and FTIR studies. Polymer.

[13] Kishi A., Tanaka M., Mochizuki A. (2009). Comparative study on water structures in PolyHEMA and PolyMEA by XRD-DSC simultaneous measurement. J. Appl. Polym. Sci..

[14] Hatakeyama H., Hatakeyama T. (1998). Interaction between water and hydrophilic polymers. Thermochim. Acta.

[15] Tanaka M. (2000). Cold crystallization of water in hydrated poly(2-methoxyethyl acrylate) (PMEA). Polym. Int..

[16] Shibukawa M., Aoyagi K., Sakamoto R., Oguma K. (1999). Liquid chromatography and differential scanning calorimetry studies on the states of water in hydrophilic polymer gel packings in relation to retention selectivity. J. Chromatogr. A.

[17] Wan L.S., Xu Z.K., Huang X.J., Wang Z.G., Ye P. (2005). Hemocompatibility of poly(acrylonitrile-co-N-vinyl-2-pyrrolidone)s: Swelling behavior and water states. Macromol. Biosci..

[18] Miwa Y., Ishida H., Saitô H., Tanaka M., Mochizuki A. (2009). Network structures and dynamics of dry and swollen poly(acrylate)s. Characterization of high- and low-frequency motions as revealed by suppressed or recovered intensities (SRI) analysis of 13C NMR. Polymer.

[19] Morita S., Tanaka M., Ozaki Y. (2007). Time-resolved in situ ATR-IR observations of the process of sorption of water into a poly(2-methoxyethyl acrylate) film. Langmuir.

[20] Zhou J., Wang X., Wang Y., Huang G., Yang X., Zhang Y., Xiong Y., Lui L., Zhao X., Fu W. (2021). A novel THz molecule-selective sensing strategy in aqueous environments: THz-ATR spectroscopy integrated with a smart hydrogel. Talanta.

[21] Van Den Bulcke A.I., Bogdanov B., De Rooze N., Schacht E.H., Cornelissen M., Berghmans H. (2000). Structural and Rheological Properties of Methacrylamide Modified Gelatin Hydrogels. Biomacromolecules.

[22] Gorgieva S., Kokol V. (2011). Collagen-vs. Gelatin-Based Biomaterials and Their Biocompatibility: Review and Perspectives. Biomater. Appl. Nanomed..

[23] Ahadian S., Ramón-Azcón J., Estili M., Obregón R., Shiku H., Matsue T. (2014). Facile and rapid generation of 3D chemical gradients within hydrogels for high-throughput drug screening applications. Biosens. Bioelectron..

[24] Kaemmerer E., Melchels F.P.W., Holzapfel B.M., Meckel T., Hutmacher D.W., Loessner D. (2014). Gelatine methacrylamide-based hydrogels: An alternative three-dimensional cancer cell culture system. Acta Biomater..

[25] Nichol J.W., Koshy S.T., Bae H., Hwang C.M., Yamanlar S., Khademhosseini A. (2010). Cell-laden microengineered gelatin methacrylate hydrogels. Biomaterials.

[26] Chen Y.C., Lin R.Z., Qi H., Yang Y., Bae H., Melero-Martin J.M., Khademhosseini A. (2012). Functional human vascular network generated in photocrosslinkable gelatin methacrylate hydrogels. Adv. Funct. Mater..

[27] Yang J., Zhang Y.S., Yue K., Khademhosseini A. (2017). Cell-laden hydrogels for osteochondral and cartilage tissue engineering. Acta Biomater..

[28] Santoro M., Tatara A.M., Mikos A.G. (2014). Gelatin carriers for drug and cell delivery in tissue engineering. J. Control. Release.

[29] Luo Z., Sun W., Fang J., Lee K., Li S., Gu Z., Dokmeci M.R., Khademhosseini A. (2019). Biodegradable Gelatin Methacryloyl Microneedles for Transdermal Drug Delivery. Adv. Healthc. Mater..

[30] Chen P., Xia C., Mei S., Wang J., Shan Z., Lin X., Fan S. (2016). Intra-articular delivery of sinomenium encapsulated by chitosan microspheres and photo-crosslinked GelMA hydrogel ameliorates osteoarthritis by effectively regulating autophagy. Biomaterials.

[31] Modaresifar K., Hadjizadeh A., Niknejad H. (2018). Design and fabrication of GelMA/chitosan nanoparticles composite hydrogel for angiogenic growth factor delivery. Artif. Cells Nanomed. Biotechnol..

[32] Lai T.C., Yu J., Tsai W.B. (2016). Gelatin methacrylate/carboxybetaine methacrylate hydrogels with tunable crosslinking for controlled drug release. J. Mater. Chem. B.

[33] Loessner D., Meinert C., Kaemmerer E., Martine L.C., Yue K., Levett P.A., Klein T.J., Melchels F.P., Khademhosseini A., Hutmacher D.W. (2016). Functionalization, preparation and use of cell-laden gelatin methacryloyl-based hydrogels as modular tissue culture platforms. Nat. Protoc..

[34] Klotz B.J., Gawlitta D., Rosenberg A.J.W.P., Malda J., Melchels F.P.W. (2016). Gelatin-Methacryloyl Hydrogels: Towards Biofabrication-Based Tissue Repair. Trends Biotechnol..

[35] Yue K., Li X., Schrobback K., Sheikhi A., Annabi N., Leijten J., Zhang W., Zhang Y.S., Hutmacher D.W., Klein T.J. (2017). Structural analysis of photocrosslinkable methacryloyl-modified protein derivatives. Biomaterials.

[36] Yue K., Trujillo-de Santiago G., Alvarez M.M., Tamayol A., Annabi N., Khademhosseini A. (2015). Synthesis, properties, and biomedical applications of gelatin methacryloyl (GelMA) hydrogels. Biomaterials.

[37] Bakravi A., Ahamadian Y., Hashemi H., Namazi H. (2018). Synthesis of gelatin-based biodegradable hydrogel nanocomposite and their application as drug delivery agent. Adv. Polym. Technol..

[38] Zhu M., Wang Y., Ferracci G., Zheng J., Cho N.J., Lee B.H. (2019). Gelatin methacryloyl and its hydrogels with an exceptional degree of controllability and batch-to-batch consistency. Sci. Rep..

[39] Yoon H.J., Shin S.R., Cha J.M., Lee S.-H., Kim J.-H., Do J.T., Song H., Bae H. (2016). Cold Water Fish Gelatin Methacryloyl Hydrogel for Tissue Engineering Application. PLoS ONE.

[40] Shie M.Y., Lee J.J., Ho C.C., Yen S.Y., Ng H.Y., Chen Y.W. (2020). Effects of gelatin methacrylate bio-ink concentration on mechano-physical properties and human dermal fibroblast behavior. Polymers.

[41] Jung J., Oh J. (2014). Swelling characterization of photo-cross-linked gelatin methacrylate spherical microgels for bioencapsulation. E-Polymers.

[42] Miri A.K., Hosseinabadi H.G., Cecen B., Hassan S., Zhang Y.S. (2018). Permeability mapping of gelatin methacryloyl hydrogels. Acta Biomater..

[43] Feng Y., Taraban M., Yu Y.B. (2012). The effect of ionic strength on the mechanical, structural and transport properties of peptide hydrogels. Soft Matter.

[44] Pourjavadi A., Hosseinzadeh H., Sadeghi M. (2007). Synthesis, characterization and swelling behavior of gelatin-g-poly(sodium acrylate)/kaolin superabsorbent hydrogel composites. J. Compos. Mater..

[45] Nazlı A.B., Açıkel Y.S. (2019). Loading of cancer drug resveratrol to pH-Sensitive, smart, alginate-chitosan hydrogels and investigation of controlled release kinetics. J. Drug Deliv. Sci. Technol..

[46] Vigata M., Meinert C., Hutmacher D.W., Bock N. (2020). Hydrogels as Drug Delivery Systems: A Review of Current Characterization and Evaluation Techniques. Pharmaceutics.

[47] Rezk A.I., Obiweluozor F.O., Choukrani G., Park C.H., Kim C.S. (2019). Drug release and kinetic models of anticancer drug (BTZ) from a pH-responsive alginate polydopamine hydrogel: Towards cancer chemotherapy. Int. J. Biol. Macromol..

[48] Zou X., Zhao X., Ye L., Wang Q., Li H. (2015). Preparation and drug release behavior of pH-responsive bovine serum albumin-loaded chitosan microspheres. J. Ind. Eng. Chem..

[49] Saghazadeh S., Rinoldi C., Schot M., Kashaf S.S., Sharifi F., Jalilian E., Nuutila K., Giatsidis G., Mostafalu P., Derakhshandeh H. (2018). Drug delivery systems and materials for wound healing applications. Adv. Drug Deliv. Rev..

[50] Johnson J.L., Holinej J., Williams M.D. (1993). Influence of ionic strength on matrix integrity and drug release from hydroxypropyl cellulose compacts. Int. J. Pharm..

[51] Lindahl A., Ungell A.L., Knutson L., Lennernäs H. (1997). Characterization of fluids from the stomach and proximal jejunum in men and women. Pharm. Res..

[52] Hamed R., Awadallah A., Sunoqrot S., Tarawneh O., Nazzal S., AlBaraghthi T., Al Sayyad J., Abbas A. (2016). pH-Dependent Solubility and Dissolution Behavior of Carvedilol—Case Example of a Weakly Basic BCS Class II Drug. AAPS PharmSciTech.

[53] Hamed R. (2018). Physiological parameters of the gastrointestinal fluid impact the dissolution behavior of the BCS class IIa drug valsartan. Pharm. Dev. Technol..

[54] Fuchs A., Dressman J.B. (2014). Composition and Physicochemical Properties of Fasted-State Human Duodenal and Jejunal Fluid: A Critical Evaluation of the Available Data. J. Pharm. Sci..

[55] Lee Y.M., Kim S.Y. (2001). Stimuli-responsive interpenetrating polymer network hydrogels composed of poly(vinyl alcohol) and poly(acrylic acid). Smart Fibres, Fabrics and Clothing.

[56] Peppas N.A., Merrill E.W. (1977). Crosslinked poly(vinyl alcohol) hydrogels as swollen elastic networks. J. Appl. Polym. Sci..

[57] Canal T., Peppas N.A. (1989). Correlation between mesh size and equilibrium degree of swelling of polymeric networks. J. Biomed. Mater. Res..

[58] Schiel J.E., Hage D.S. (2005). Density measurements of potassium phosphate buffer from 4 to 45 °C. Talanta.

[59] Fels I.G. (1964). Hydration and density of collagen and gelatin. J. Appl. Polym. Sci..

[60] Fessler J.H., Hodge A.J. (1962). Ultracentrifugal observation of phase transitions in density gradients: I. The collagen system. J. Mol. Biol..

[61] Anjum F., Lienemann P.S., Metzger S., Biernaskie J., Kallos M.S., Ehrbar M. (2016). Enzyme responsive GAG-based natural-synthetic hybrid hydrogel for tunable growth factor delivery and stem cell differentiation. Biomaterials.

[62] Flory P.J., Rehner J. (1943). Statistical mechanics of cross-linked polymer networks I. Rubberlike elasticity. J. Chem. Phys..

[63] Bray J.C., Merrill E.W. (1973). Poly(vinyl alcohol) hydrogels. Formation by electron beam irradiation of aqueous solutions and subsequent crystallization. J. Appl. Polym. Sci..

[64] Gilchrist A.E., Lee S., Hu Y., Harley B.A.C. (2018). Mesenchymal stromal cell remodeling of a gelatin hydrogel microenvironment defines an artificial hematopoietic stem cell niche. bioRxiv.

[65] Ofner C.M., Bubnis W.A. (1996). Chemical and swelling evaluations of amino group crosslinking in gelatin and modified gelatin matrices. Pharm. Res..

[66] Kasapis S., Al-Marhoobi I.M., Mitchell J.R. (2003). Molecular weight effects on the glass transition of gelatin/cosolute mixtures. Biopolymers.

[67] Berg J.M., Tymoczko J.L., Stryer L. (2002). Primary Structure: Amino Acids Are Linked by Peptide Bonds to Form Polypeptide Chains. Biochemistry.

[68] Hatakeyama T., Tanaka M., Hatakeyama H. (2010). Studies on bound water restrained by poly(2-methacryloyloxyethyl phosphorylcholine): Comparison with polysaccharide-water systems. Acta Biomater..

[69] Marcus Y. (2009). Effect of ions on the structure of water: Structure making and breaking. Chem. Rev..

[70] Ganji F., Vasheghani-Farahani S., Vasheghani-Farahani E. (2010). Theoretical Description of Hydrogel Swelling: A Review. www.SID.ir.

[71] Yang X., Dargaville B.L., Hutmacher D.W. (2021). Elucidating the Molecular Mechanisms for the Interaction of Water with PEG-based Hydrogels: Influence of Ionic Strength and Gel Network Structure. Polymers.

[72] Horkay F., Tasaki I., Basser P.J. (2000). Effect of monovalent-divalent cation exchange on the swelling of polyacrylate hydrogels in physiological salt solutions. Biomacromolecules.

[73] Cavallo A., Madaghiele M., Masullo U., Lionetto M.G., Sannino A. (2017). Photo-crosslinked poly(ethylene glycol) diacrylate (PEGDA) hydrogels from low molecular weight prepolymer: Swelling and permeation studies. J. Appl. Polym. Sci..

[74] Vigata M., Meinert C., Pahoff S., Bock N., Hutmacher D.W. (2020). Gelatin methacryloyl hydrogels control the localized delivery of albumin-bound paclitaxel. Polymers.

[75] Park J.E., Barbul A. (2004). Understanding the role of immune regulation in wound healing. Am. J. Surg..

[76] Bukhari S.M.H., Khan S., Rehanullah M., Ranjha N.M. (2015). Synthesis and Characterization of Chemically Cross-Linked Acrylic Acid/Gelatin Hydrogels: Effect of pH and Composition on Swelling and Drug Release. Int. J. Polym. Sci..

[77] Hollingshead S., Liu J.C. (2020). pH-Sensitive Mechanical Properties of Elastin-Based Hydrogels. Macromol. Biosci..

[78] Shirahama H., Lee B.H., Tan L.P., Cho N.J. (2016). Precise tuning of facile one-pot gelatin methacryloyl (GelMA) synthesis. Sci. Rep..

[79] Van Vlierberghe S., Graulus G.J., Samal S.K., Van Nieuwenhove I., Dubruel P. (2014). Porous hydrogel biomedical foam scaffolds for tissue repair. Biomedical Foams for Tissue Engineering Applications.

[80] Alihosseini F. (2016). Plant-based compounds for antimicrobial textiles. Antimicrobial Textiles.

[81] Liang J., Grijpma D.W., Poot A.A. (2020). Tough and biocompatible hybrid networks prepared from methacrylated poly(trimethylene carbonate) (PTMC) and methacrylated gelatin. Eur. Polym. J..

[82] Percot A., Lafleur M., Zhu X.X. (2000). New hydrogels based on N-isopropylacrylamide copolymers crosslinked with polylysine: Membrane immobilization systems. Polymer.

[83] Griset A.P., Walpole J., Liu R., Gaffey A., Colson Y.L., Grinstaff M.W. (2009). Expansile nanoparticles: Synthesis, characterization, and in vivo efficacy of an acid-responsive polymeric drug delivery system. J. Am. Chem. Soc..

[84] Sadeghi M., Mohammadinasab E., Shafiei F. (2012). Synthesis and Investigation of a Novel pH-and Salt-Responsive Superabsorbent Hydrogel Based on Pectin. Curr. World Environ..

[85] Cefazolin Sodium C_14_H_13_N_8_NaO_4_S_3_-PubChem. https://pubchem.ncbi.nlm.nih.gov/compound/cefazolin_sodium#section=Top.

[86] Shah S.R., Henslee A.M., Spicer P.P., Yokota S., Petrichenko S., Allahabadi S., Bennett G., Wong M.E., Kasper F.K., Mikos A.G. (2014). Effects of antibiotic physicochemical properties on their release kinetics from biodegradable polymer microparticles. Pharm. Res..

[87] Li J., Mooney D.J. (2016). Designing hydrogels for controlled drug delivery. Nat. Rev. Mater..

