# Supplementary material for: Deciphering the Molecular Mechanism of Water Interaction with Gelatin Methacryloyl Hydrogels: Role of Ionic Strength, pH, Drug Loading and Hydrogel Network Characteristics
Source: Biomedicines. 2021 May 19;9(5):574. doi: 10.3390/biomedicines9050574 (PMC8161260; doi:10.3390/biomedicines9050574)
Supplement: Supplementary file 1 [file biomedicines-09-00574-s001.zip › biomedicines-1194270-supplementary materials.pdf]

Supplementary Materials

Deciphering the Molecular mechanism for the Interaction of Water with Gelatin Methacryloyl Hydrogels: Role of Ionic Strength, pH, Drug Loading, and the Hydrogel Network Characteristics

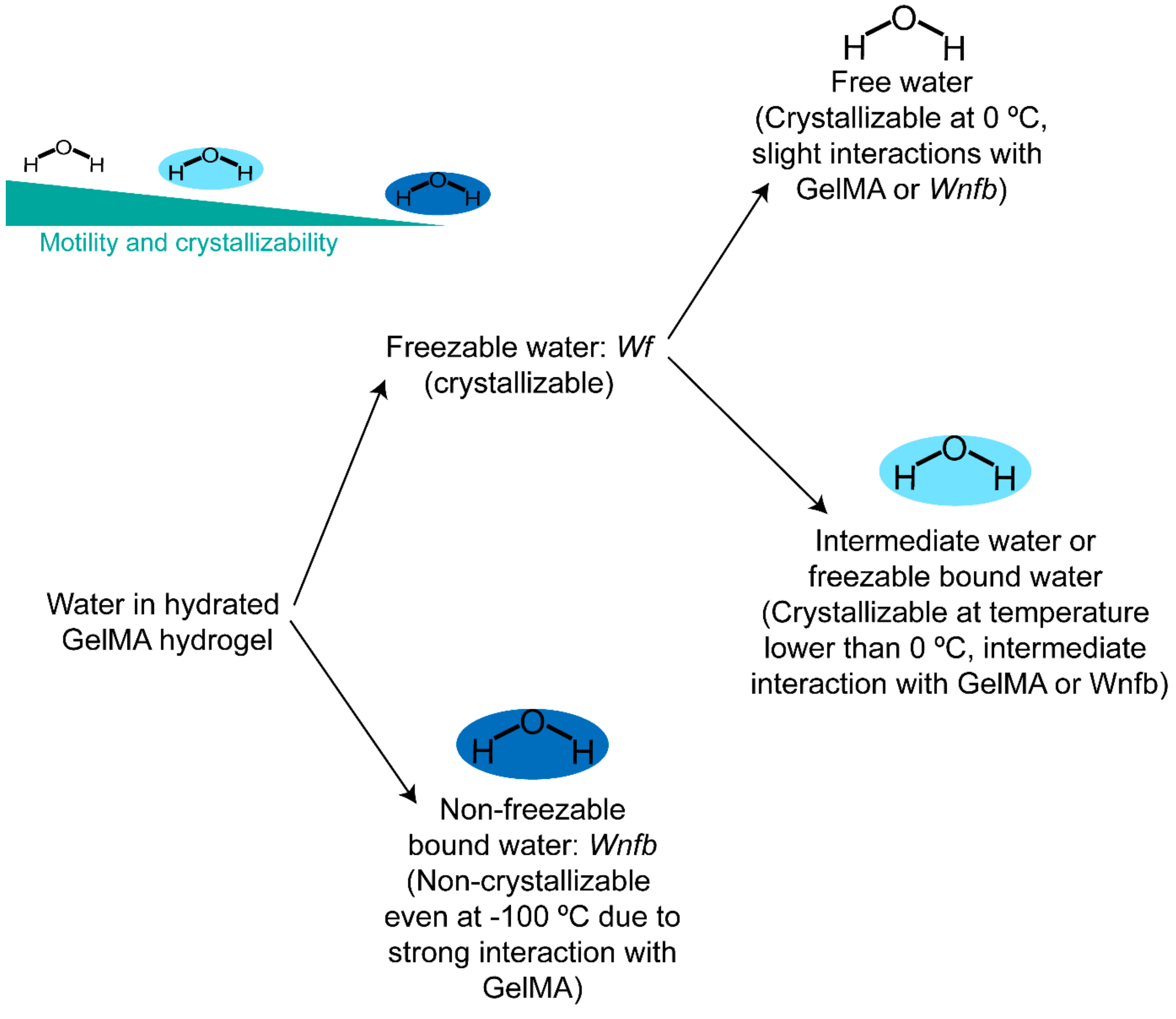

Figure S1. The different water states in hydrated GelMA hydrogels: Non-freezable bound water, freezable bound water (intermediate water), and free water. Modified from [1]. Differential scanning calorimetry (DSC) thermogram of 15% GelMA crosslinked in PBS and swelled in PBS.

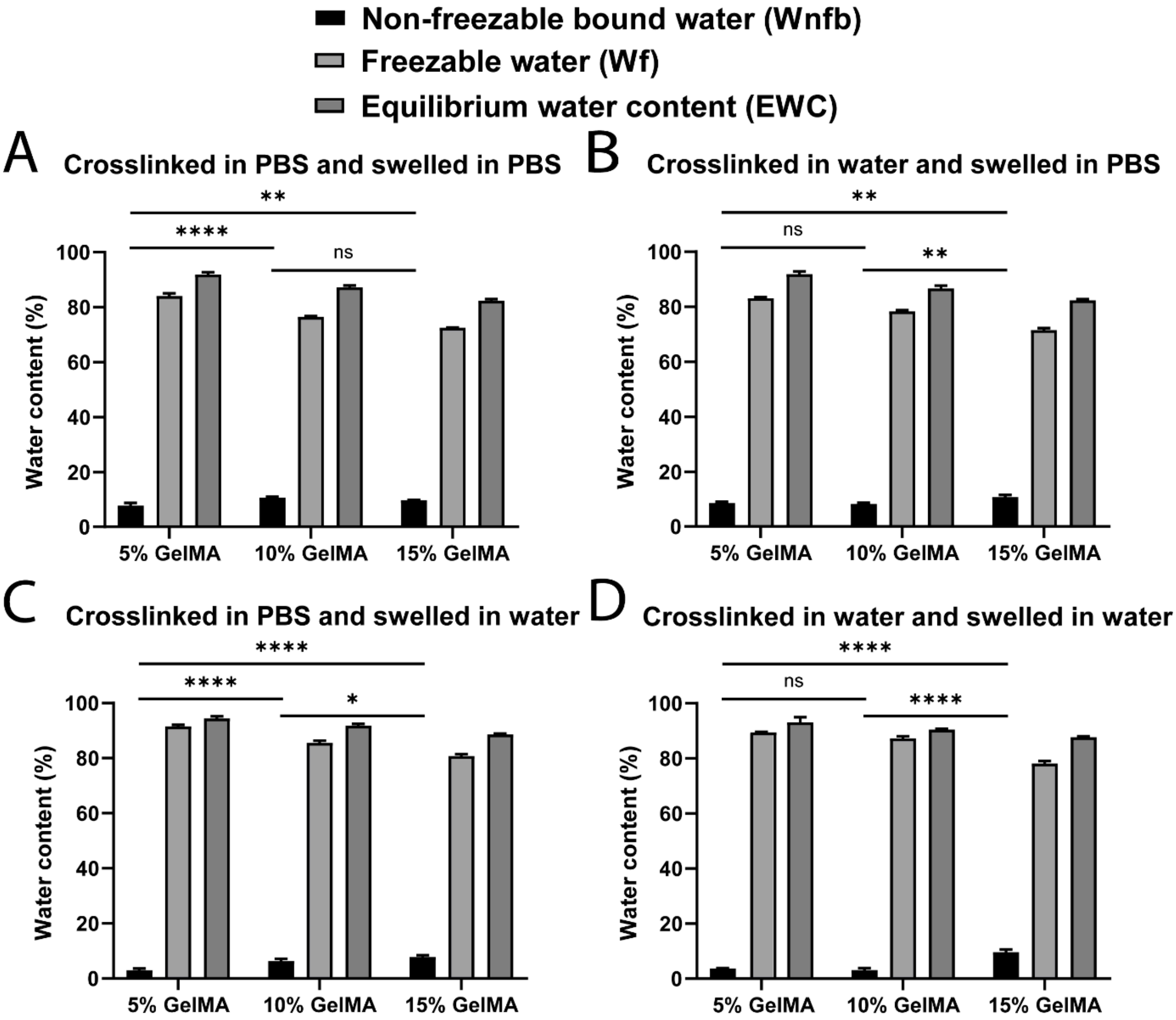

Figure S2. Water content for GelMA hydrogels (5% to 15%) crosslinked and swelled in different media. Non-freezable bound water (W_{nfb}), freezable water (W_f) and equilibrium water content (EWC) of GelMA hydrogel (**A**) crosslinked and swelled in PBS, (**B**) crosslinked in water and swelled in PBS, (**C**) crosslinked in PBS and swelled in water, and (**D**) cross-linked and swelled in water ($n = 5$ for EWC; $n = 3$ for W_{nfb} and W_f). Ns = non significant. * = $p < 0.05$. ** = $p < 0.01$. **** = $p < 0.0001$.

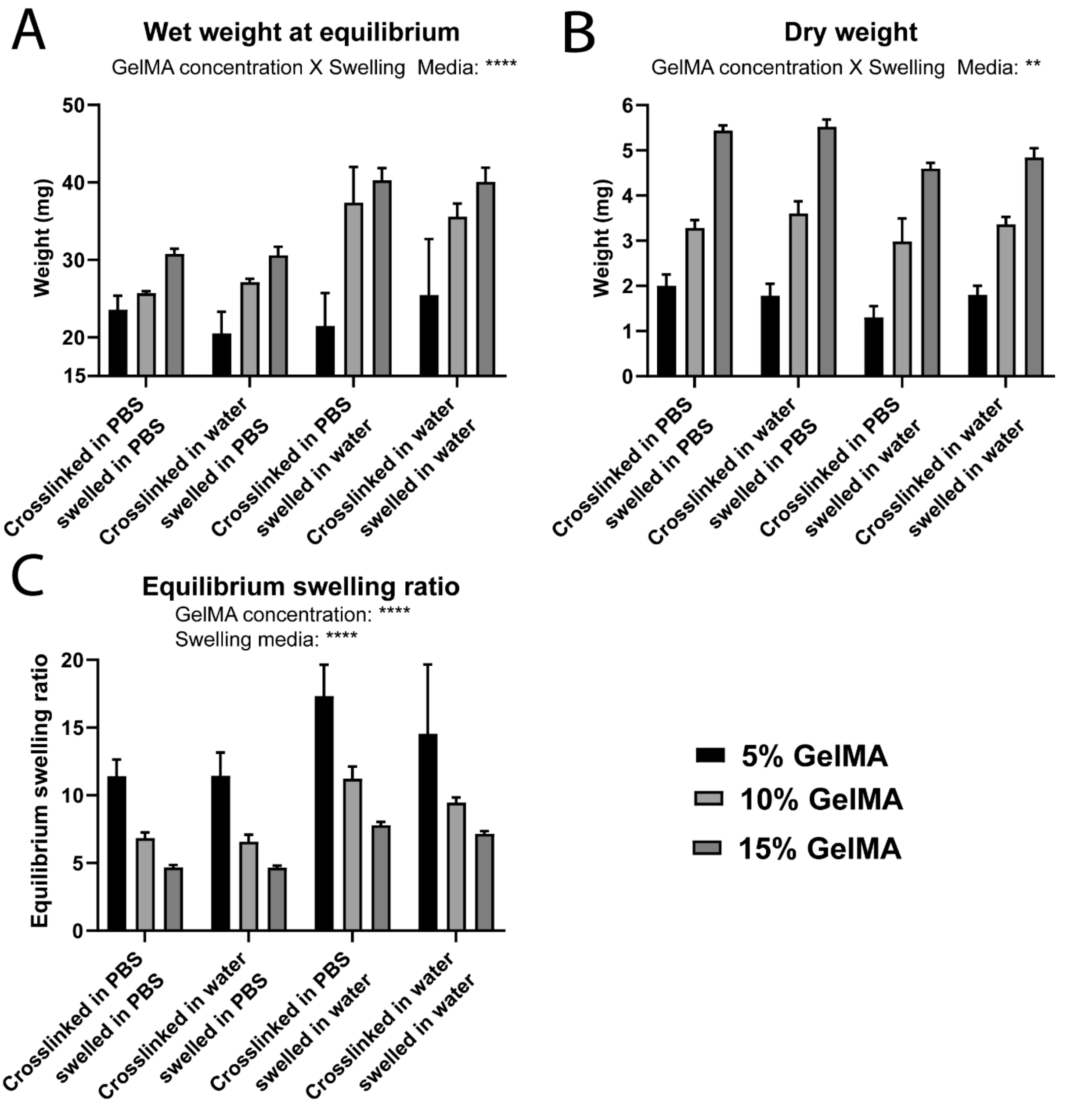

Figure S3. Wet weight, dry weight and equilibrium swelling ratio for GelMA hydrogels crosslinked and swelled in different media. (**A**) Wet weight, (**B**) Dry weight, (**C**) Equilibrium swelling ratio for GelMA hydrogels (5% to 15% gel fraction) crosslinked and swelled in different media.

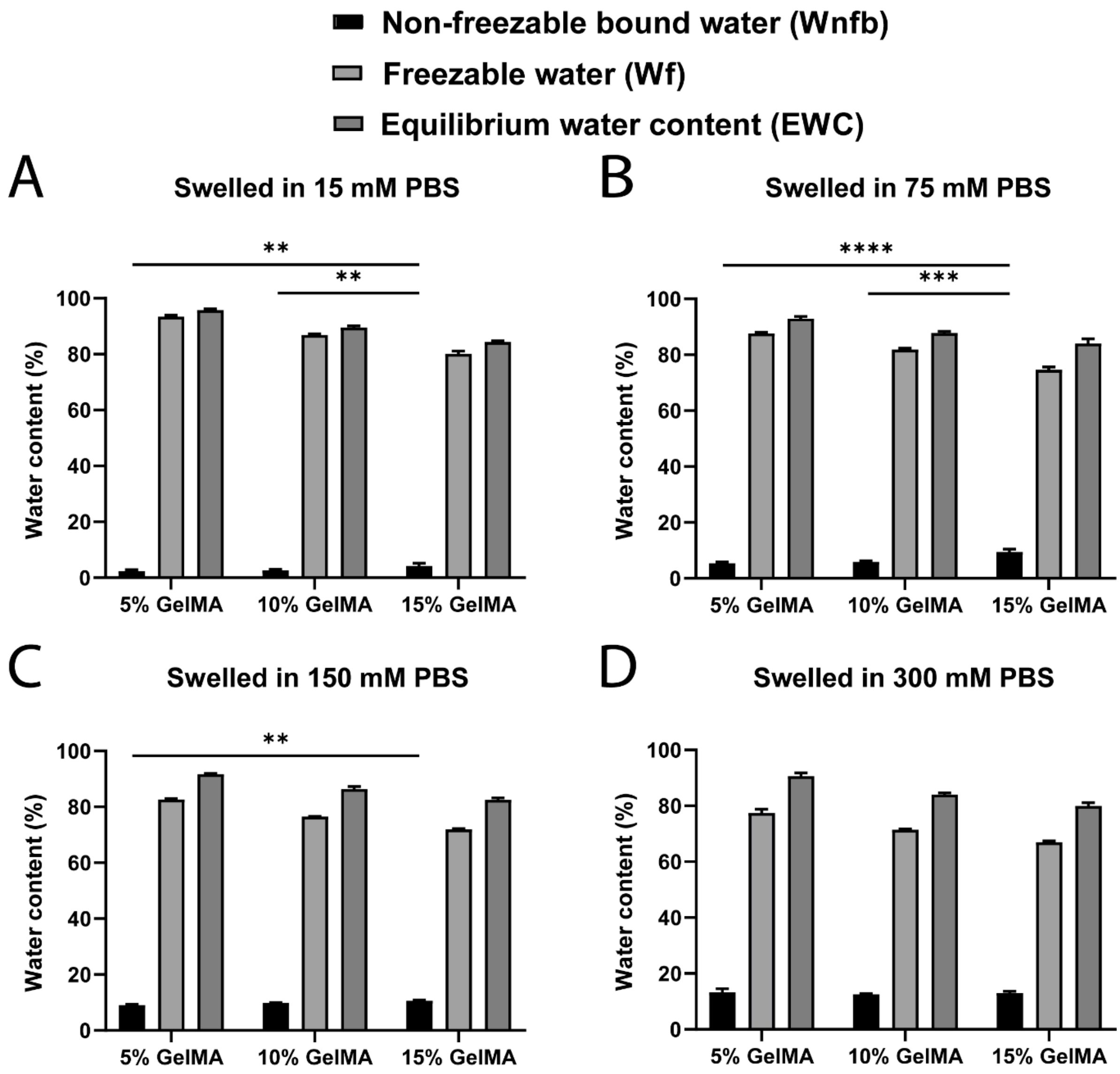

Figure S4. Water content for GelMA hydrogels (5% to 15% gel fraction) swelled in media with increasing ionic strength. (**A**) GelMA hydrogels were crosslinked in water and swelled in PBS at 0.1× = 15 mM. (**B**) GelMA hydrogels were cross-linked in water and swelled in PBS at 0.5× = 75 mM. (**C**) GelMA hydrogels were crosslinked in water and swelled in PBS at 1× = 150 mM. (**D**) GleMA hydrogels were crosslinked in water and swelled in PBS 2× = 300 mM. Equilibrium water content (EWC, $n = 5$), non-freezable bound water (W_{nfb}, $n = 3$), and freezable water (W_f, $n = 3$). Ns = non-significant. ** = $p < 0.01$. *** = $p < 0.001$. **** = $p < 0.0001$.

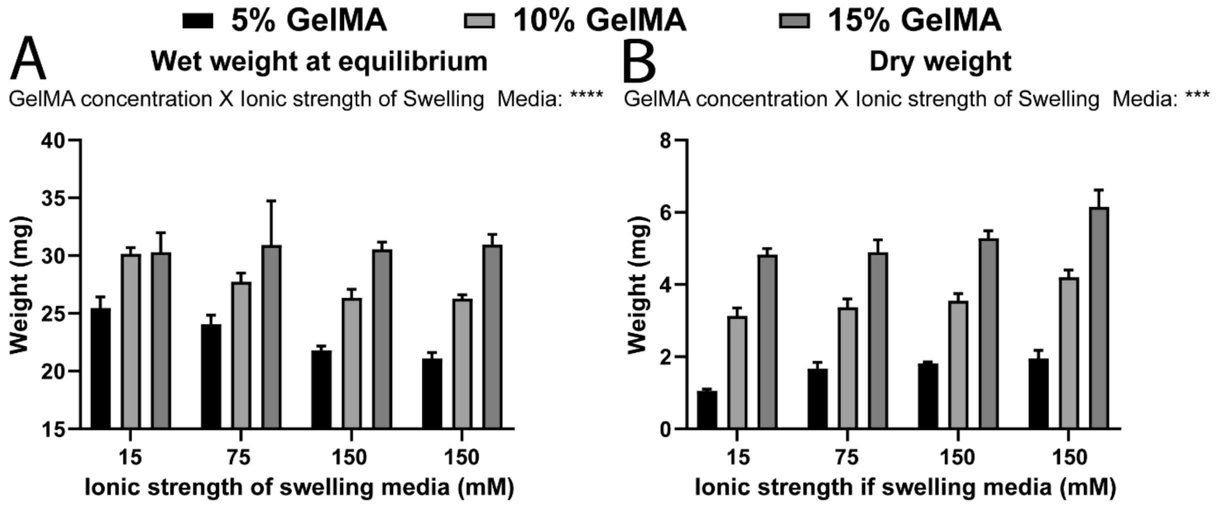

Figure S5. Wet weight and dry weight for GelMA hydrogels swelled in different ionic strengths. (**A**) Wet weight, (**B**) Dry weight, for GelMA hydrogels (5% to 15% gel fraction) swelled in different ionic strengths.

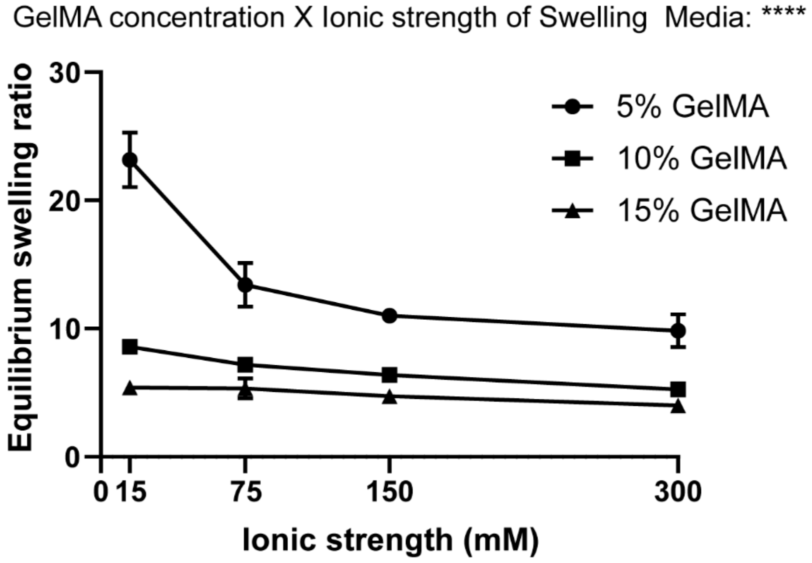

Figure S6. Equilibrium swelling ratio for GelMA hydrogels (5% to 15%) crosslinked in water and swelled at different ionic strengths.

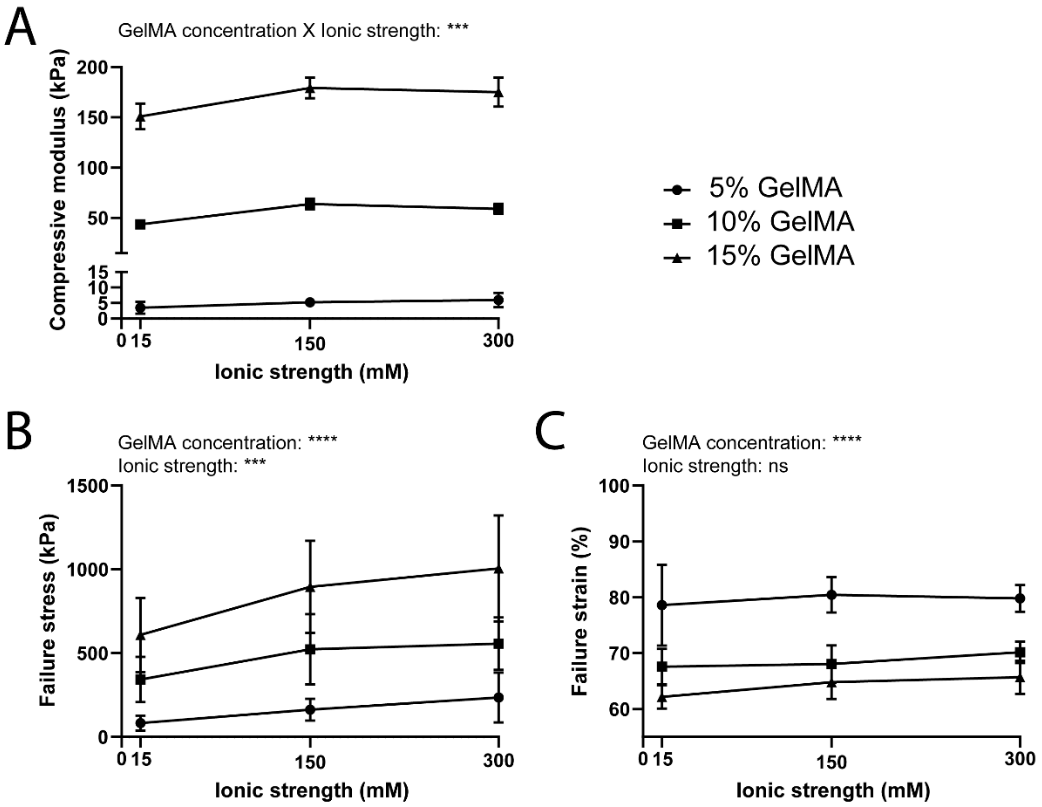

Figure S7. Mechanical properties for GelMA hydrogels (5% to 15% gel fraction) swelled at different ionic strength. (**A**) Compressive modulus. (**B**) Failure stress. (**C**) Failure strain. Data are shown as means ± standard deviation, *n* = 6–8.

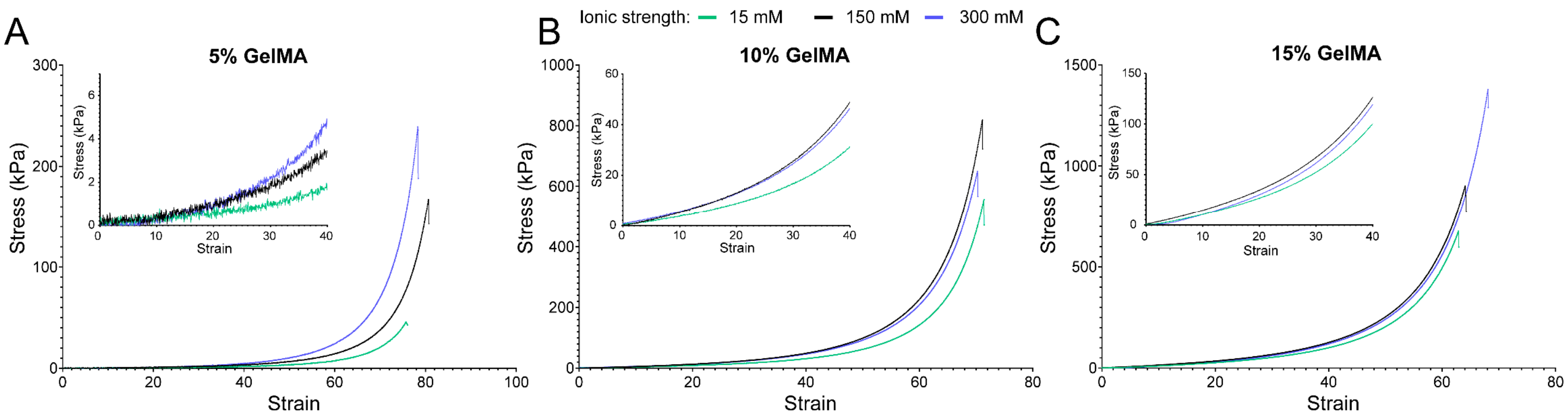

Figure S8. Rerpesentative stress-strain curves for (**A**) 5%, (**B**) 10%, and (**C**) 15% GelMA hydrogels crosslinked in water and swelled at different ionic strengths.

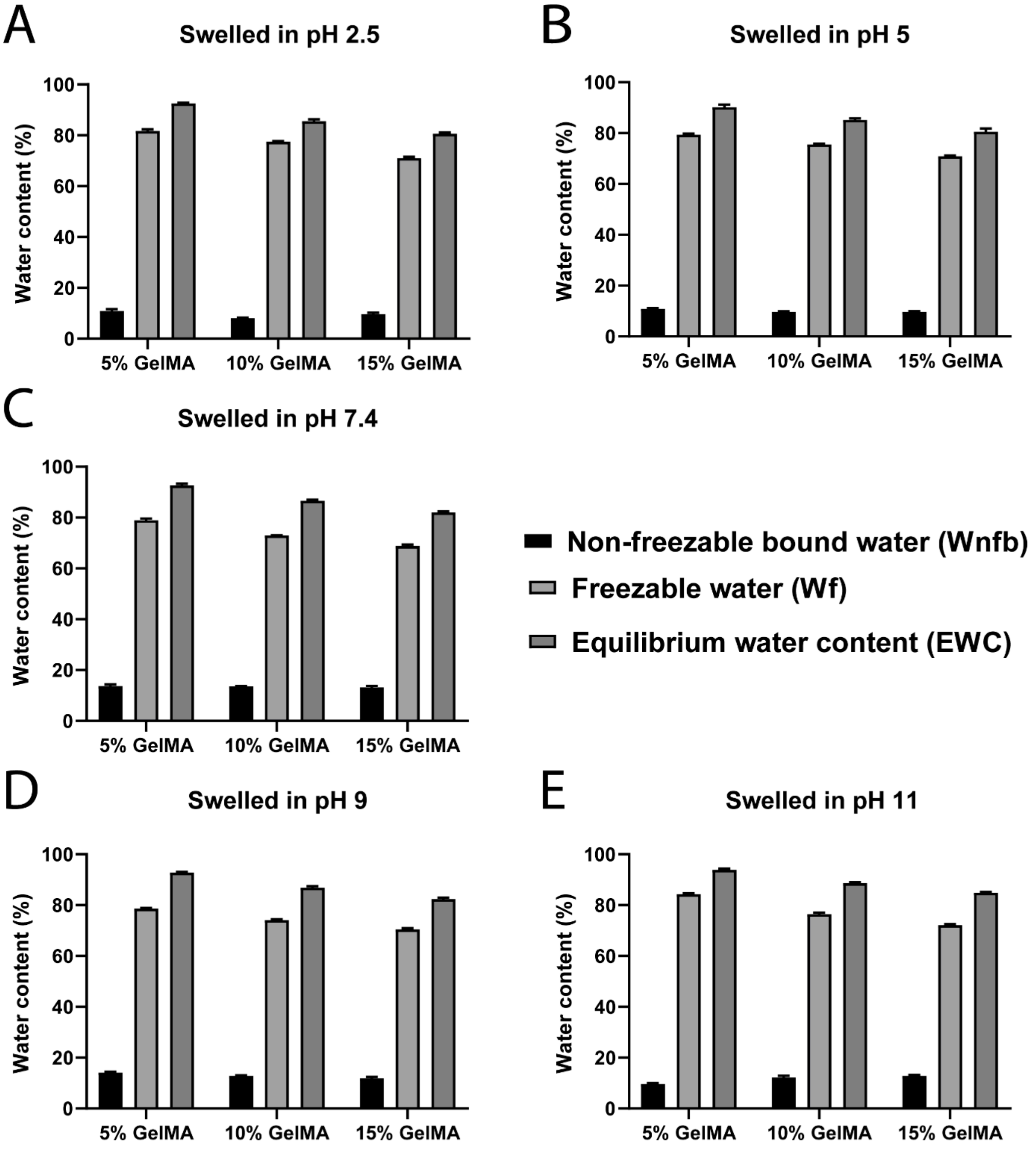

Figure S9. Water content for GelMA hydrogels (5% to 15% gel fraction) crosslinked in water and swelled in PBS at different pH. (**A**) pH 2.5. (**B**) pH 5. (**C**) pH 7.4. (**D**) pH 9. (**E**) pH 11. Equilibrium water content (EWC, n = 5), non-freezable bound water (W_{nfb}, n = 3), and freezable water (W_f, n = 3).

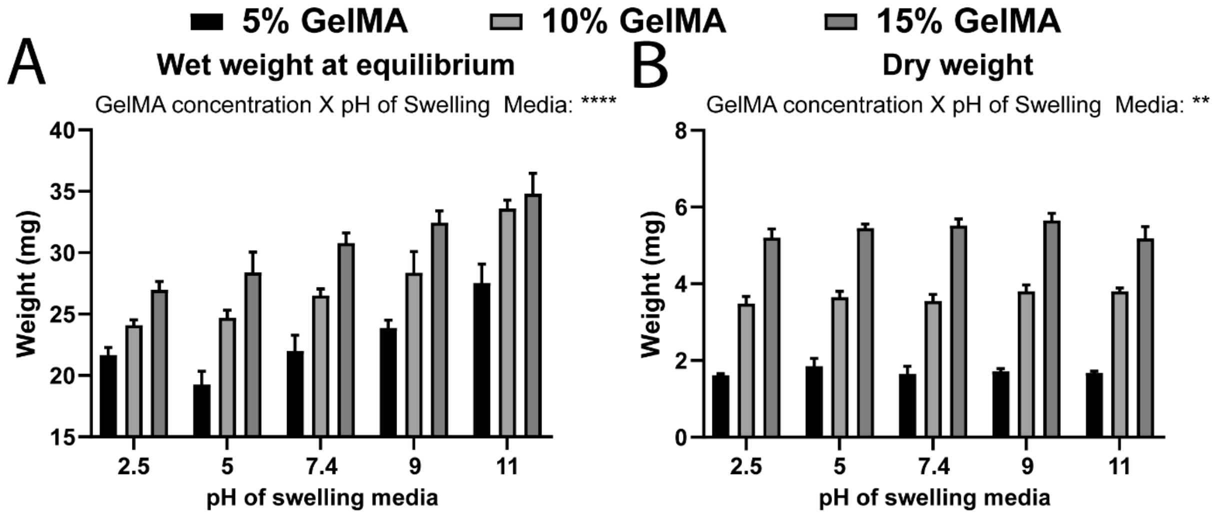

Figure S10. Wet weight and dry weight for GelMA hydrogels swelled in different pH. (**A**) Wet weight, (**B**) Dry weight, for GelMA hydrogels (5% to 15% gel fraction) swelled in different pH.

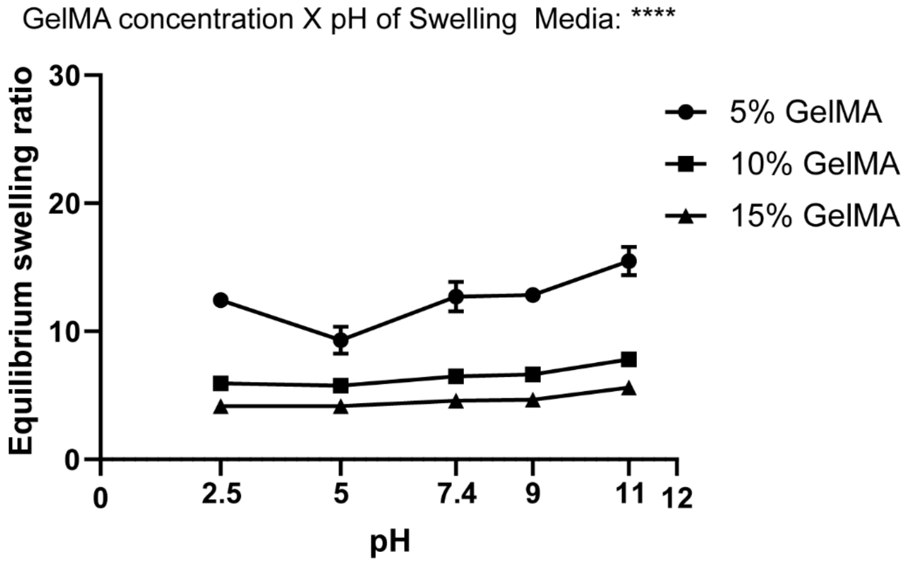

Firgure S11. Equilibrium swelling ratio for GelMA hydrogels (5 to 15%) crosslinked in water and swelled at different pH.

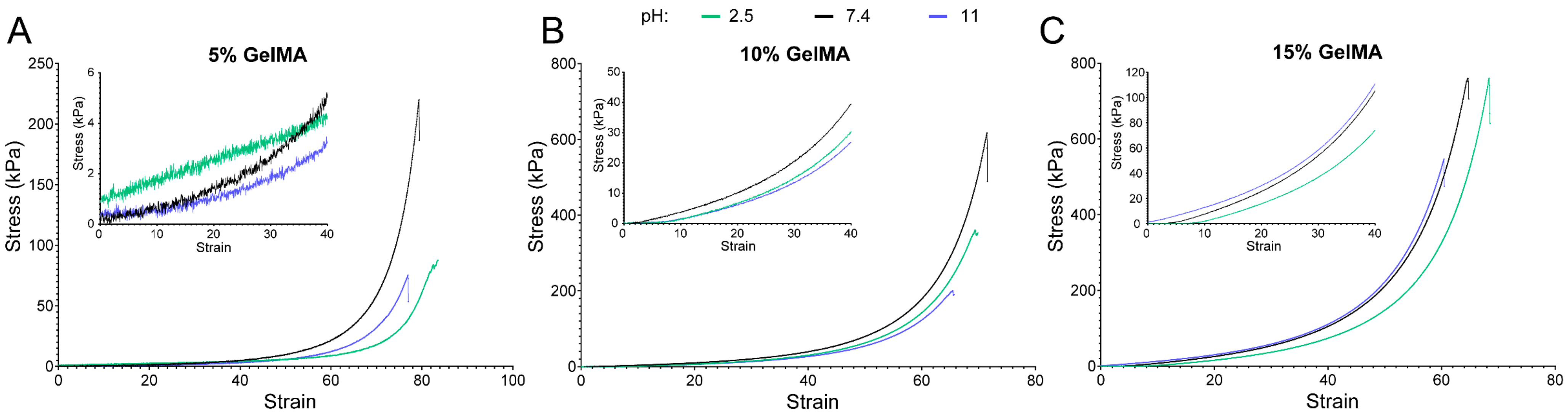

Figure S12. Representative stress-strain curves for (**A**) 5%, (**B**) 10%, and (**C**) 15% GelMA hydrogels crosslinked in water and swelled at different pH.

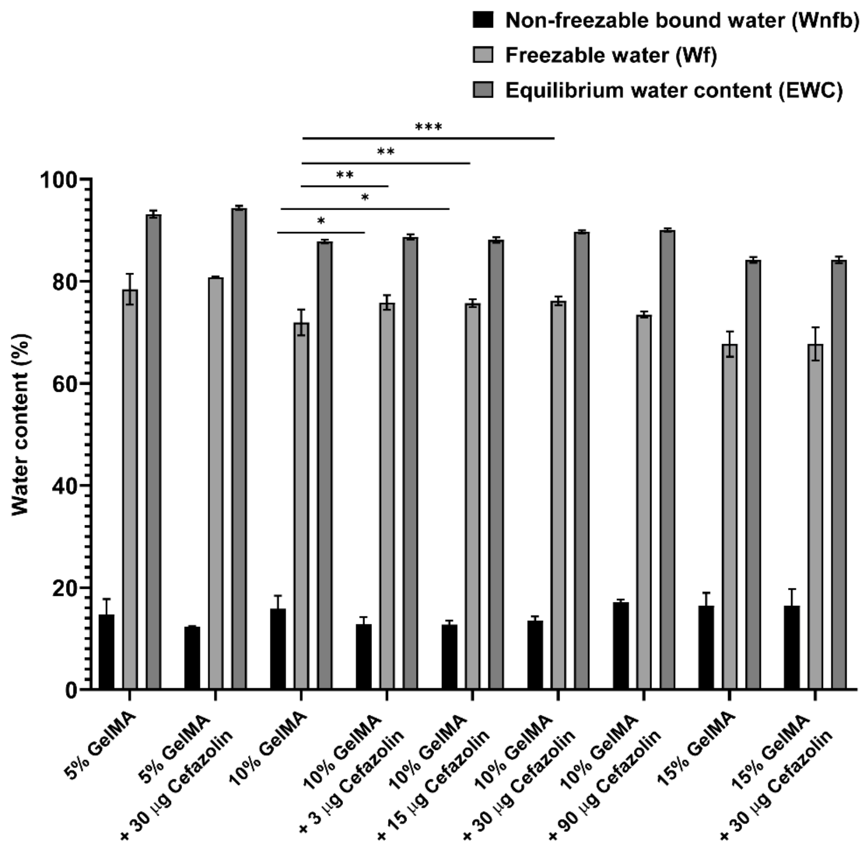

Figure S13. Water content for GelMA hydrogels (5% to 15% gel fraction) crosslinked with 0, 3, 15, 30, or 90 μg cefazolin in PBS. Hydrogels were analyzed immediately after crosslinking. Equilibrium water content (EWC, $n = 5$), non-freezable bound water (W_{nfb}, $n = 3$), and freezable water (W_f, $n = 3$). * = $p < 0.05$. ** = $p < 0.01$.

References

1. Tanaka, M.; Hayashi, T.; Morita, S. The roles of water molecules at the biointerface of medical polymers. *Polym. J.* **2013**, *45*, 701–710, doi:10.1038/pj.2012.229.
